# Octopod *Hox* genes and cephalopod plesiomorphies

**DOI:** 10.1038/s41598-023-42435-0

**Published:** 2023-09-19

**Authors:** Cristian Camilo Barrera Grijalba, Sonia Victoria Rodríguez Monje, Camino Gestal, Tim Wollesen

**Affiliations:** 1 Department of Evolutionary Biology, Faculty of Life Sciences, University of Vienna, Djerassiplatz 1, 1030 Vienna, Austria; 2 Institute of Marine Research (IIM-CSIC), Eduardo Cabello 6, 36208 Vigo, Spain

**Keywords:** Evolutionary developmental biology, Developmental biology

## Abstract

Few other invertebrates captivate our attention as cephalopods do. Octopods, cuttlefish, and squids amaze with their behavior and sophisticated body plans that belong to the most intriguing among mollusks. Little is, however, known about their body plan formation and the role of *Hox* genes. The latter homeobox genes pattern the anterior–posterior body axis and have only been studied in a single decapod species so far. Here, we study developmental *Hox* and *ParaHox* gene expression in *Octopus vulgaris.* *Hox *genes are expressed in a near-to-staggered fashion, among others in homologous organs of cephalopods such as the stellate ganglia, the arms, or funnel. As in other mollusks *Hox1* is expressed in the nascent octopod shell rudiment. While *ParaHox* genes are expressed in an evolutionarily conserved fashion, *Hox* genes are also expressed in some body regions that are considered homologous among mollusks such as the cephalopod arms and funnel with the molluscan foot. We argue that cephalopod *Hox* genes are recruited to a lesser extent into the formation of non-related organ systems than previously thought and emphasize that despite all morphological innovations molecular data still reveal the ancestral molluscan heritage of cephalopods.

## Introduction

During the last decade, a wealth of studies dissected the genomic and transcriptomic machinery giving rise to the complex cephalopod body plan (e.g. Refs.^1–7^). In addition, an ever-increasing number of studies witness the amazing cognitive abilities and physiological peculiarities of Coleoida, i.e. all cephalopods but nautiluses (e.g. Refs.^8–10^). The majority of these studies emphasize the evolutionarily highly derived nature of coleoids that in certain aspects are more similar to vertebrates than to their molluscan kindship. Indeed, at first glance the cephalopod body plan looks very different compared to the generalized molluscan body plan visible in clams, snails, or tusk shells^11^. Earlier studies already inferred how the cephalopod body plan evolved from a rather sessile monoplacophoran-like ancestor into a motile organism that conquered the pelagic realm by elongation of the dorso-ventral body axis and reduction of the external shell^12^. This evolutionary scenario included the morphological transition of the molluscan foot into an arm crown and a funnel that were used to quickly navigate through the 3-dimensional pelagic realm and allowed cephalopods to unlock new dietary resources. Surprisingly little is however still known about the formation of the cephalopod body plan on a molecular level, and only few studies were concerned with molecular pathways and genes that establish the anterior–posterior (AP) and dorsal–ventral (DV) body axes^13,14^. *Hox* genes have been shown to be involved in the regulatory network that establishes the AP axis in bilaterians^15^. They are well characterized DNA sequences that encode for a group of homeotic transcription factors related to regulation of tissue formation and structure spatial organization in the embryos during early development^16^. In addition to these functions, there is evidence indicating association between the *Hox* genes and pathways that establish cell types^17^. *Hox* genes are present across the Metazoa, and are often associated with the tremendous diversity of body plans. In terms of structure, *Hox* genes are defined by a region in their sequence known as the homeobox. This sequence encodes for the homeodomain, responsible for the DNA-binding property of the *Hox* transcription factors^18^. Moreover, these homeobox-containing genes are orthologs of members of the *Hox* cluster present in mammals and *Drosophila melanogaster*^19^. Regulation mediated by *Hox* genes at the transcriptional level is the result of the interaction between the *Hox* transcription factors with regulatory complexes made up of cofactors with DNA-binding domains that increase the specificity of the interaction, and DNA non-binding independent factors which may serve as stabilizers of this complex^20^. However, there is also evidence that the regulatory process can take place in the absence of cofactor proteins in *Drosophila melanogaster*^21^. Interestingly, *Hox*-mediated regulation by posttranscriptional activity has also been evidenced^22^.

The regulatory effect of the *Hox* transcription factors can either suppress or activate the expression of the target gene, and these interactions can occur in a spatial and temporal fashion during development^15^. This fact is related to their organization in clusters on the chromosome level within the genome. *Hox* genes are classified in anterior (*Hox1-5*), central (*Lox2,4, 5,* and *Hox7*) and posterior (*Post1-2*) groups^23,24^. Remotely related bilaterians such as fruit fly, roundworm, mouse or lancelet, show collinear *Hox* expression, i.e. *Hox* genes are expressed in a staggered fashion along the developing AP-axis matching their organization in the genome^25^. In addition, temporal collinearity has been observed in some organisms, i.e. anterior genes of the *Hox* cluster are expressed earlier during ontogeny than genes positioned at the posterior end of the cluster^25^. The number and arrangement of *Hox* genes, however, differ among organismal groups. For instance, vertebrates exhibit four *Hox* clusters due to genome duplication events^22^.

**Figure 1:**
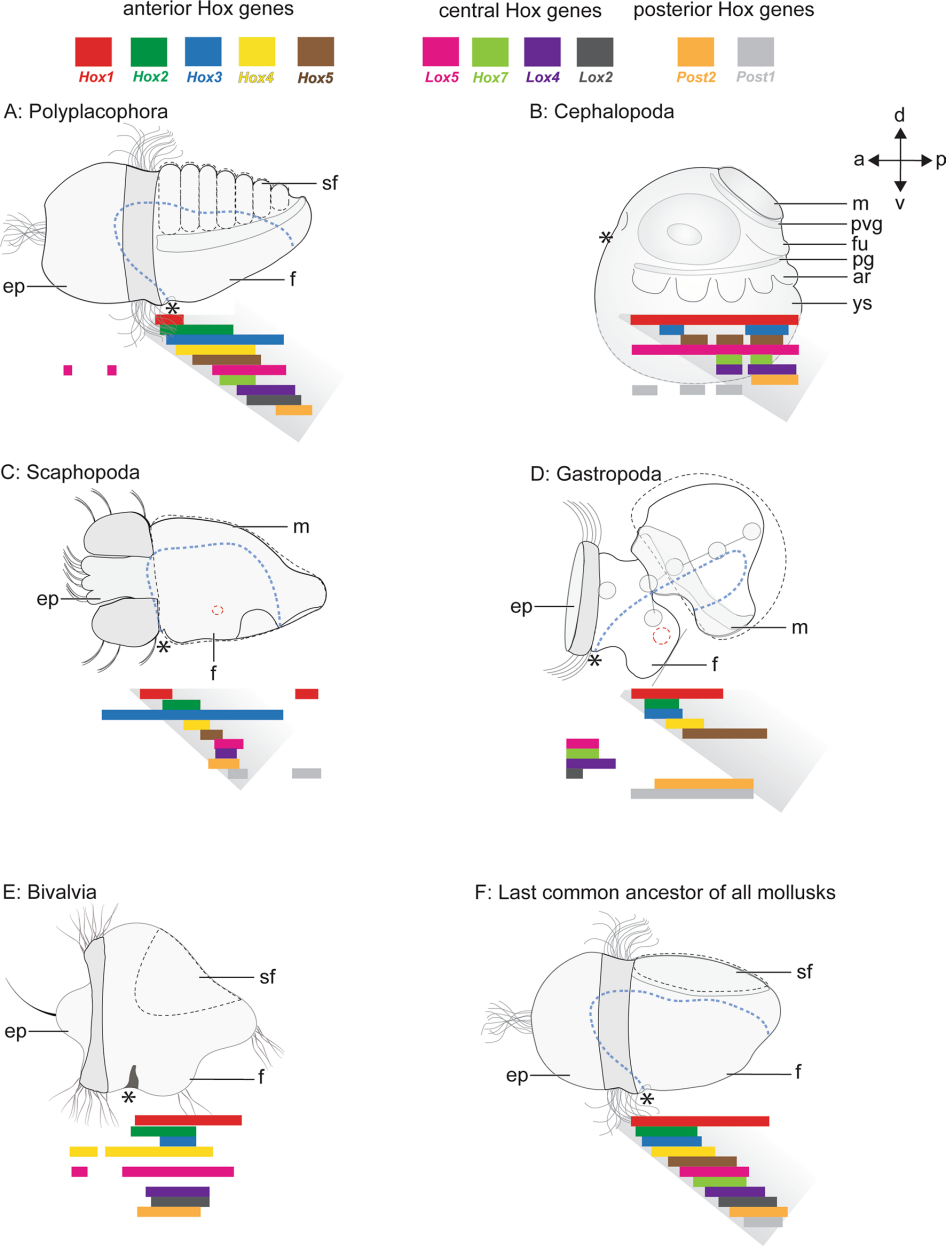
*Hox* gene expression in molluscan developmental stages. Dorsal (d)–ventral (v) and anterior (a)–posterior (P) axes indicate the orientation (all lateral views). Staggered expression is present in polyplacophorans (**A**) and gastropods (**D**). The decapod cephalopod *Euprymna scolopes* (**B**)^13^ and the gastropod *Gibbula varia* depicted herein (**D**) show traces of staggered *Hox* expression^32^, however recently, Huan et al.^35^ evidenced staggered *Hox* expression in the gastropod *Lottia goshimai* (not shown). The early mid-stage trochophore of the scaphopod *Antalis entalis* exhibits near-to-staggered *Hox* expression (**C**)^34^, while no staggered expression was observed in bivalve embryos so far (**E**)^36^. The above-mentioned data and the presence of staggered *Hox* expression in other bilaterians suggests staggered *Hox* expression in the last common ancestor of all mollusks (**F**). The shell/shell plates (dashed black lines), the prototroch/velum (shaded in dark gray), the digestive tract (stippled blue lines), and the blastopore/mouth (asterisks) are outlined. *a anterior, ar* arm, *d* dorsal, *ep* episphere, *f* foot, *fu* funnel, *m* mantle, *v* ventral, *p* posterior, *pg* pedal ganglion, *pvg* palliovisceral ganglion, *sf* shell field, *ys* yolk sac. Sketch modified from Ref.^26^.

Among Spiralia, a clade composed of mollusks, annelids, bryozoans, nemerteans, chaetognaths and other organisms, staggered and non-staggered *Hox* expression has been observed^26–28^. When the first *Hox* genes were reported for mollusks, it appeared that they were primarily recruited into the evolution of morphological novelties, rather than being expressed in a staggered fashion in the nervous system along the AP-axis^13,29–32^ (Fig. 1). It was not until other phylogenetically informative taxa such as the aculiferan polyplacophorans and the conchiferan scaphopods were observed that traces of staggered *Hox* expression were found^33,34^ (Fig. 1A,C). After reanalyzing the previously published *Hox* gene expression dataset of the decapod cephalopod *Euprymna scolopes*^13^ and the gastropod *Gibbula varia*^32^, Wollesen et al. also found traces of staggered *Hox* expression (Fig. 1B,D ^34^). An important route to find this staggered expression was to study different developmental stages since *Hox* genes may be expressed in various domains during ontogeny which also a subsequent study on a gastropod revealed^35^. While staggered *Hox* expression has not (yet) been reported for bivalves (Fig. 1E), the aplacophoran solenogastres and caudofoveates as well as monoplacophorans remain still unstudied^36^.

Another evolutionarily highly conserved cluster of genes, the *ParaHox* genes, has been suggested to mainly pattern the bilaterian digestive system, however, *Xlox*, *Caudal*, and *Gsx* appear to also pattern other organs in a variety of animals^37,38^. *ParaHox* genes are thought to have belonged to a putative ancestral *Hox* cluster that separated during evolution.

Here, we study *Hox* genes and *ParaHox* genes in two mid-embryonic stages of *Octopus vulgaris,* a representative of the hitherto unstudied octopod clade. We show that *Hox* genes show signs of staggered expression, and we reveal that *Hox1* is also expressed in the anlage of the shell field. Our study contributes new expression patterns of *Hox* genes (*Hox1, Hox3, Hox5, Lox4 & Lox2*) and *ParaHox* genes (*Gsx* and *Xlox)* and our data suggest that cephalopods show clear similarities, also on the molecular level, with their molluscan relatives.

## Material and methods

### Octopus husbandry

Adult octopuses for this study were caught by artisanal fishermen off the shore of Vigo, Spain (42° 13′ 43″ N 8° 48′ 44″ W) and maintained in a 400 L flow-through system tank according to Iglesias et al.^39^.

PVC shelters were provided as refuges to induce natural spawning. Individuals were maintained under standard conditions of summer natural photoperiod, seawater temperatures (19–23 °C) and they were fed *ad libitum *with thawed crabs and fish three days a week. Once egg laying occurred, the female was kept in a separate tank at the same water temperature. The female took care of the eggs without being fed until the offspring hatching. *Octopus* prehatching embryos were collected at different time-points and staged according to Naef^12^.

### Animal collection and fixation

Individuals of the developmental stages VIII, XI, XV, XVIII, and XX of *O. vulgaris* were anaesthetized using cold seawater (less than 2 °C) and their egg capsules were carefully punctured with a needle. Animals were fixed in 4% PFA in MOPS buffer, washed and stored in ice-cold 100% methanol at − 20 °C as described earlier for subsequent in situ hybridization experiments (see Wollesen et al. 2014 for details on fixation procedure)^40^. More individuals of the same developmental stages were transferred to RNAlater (Life Technologies, Vienna, Austria) for subsequent RNA extraction. After 1 h at 4 °C, samples were stored at − 20 °C. RNA was extracted using a RNA extraction kit (Qiagen, Roermond, Netherlands) and stored at − 80 °C.

All animal experiments were performed according to the Spanish law RD53/2013 within the framework of European Union directive on animal welfare (Directive 2010/63/EU) for the protection of animals employed for experimentation and other scientific purposes, following the Guidelines for the care and welfare of cephalopods published by Fiorito et al.^41^. In the present study, only octopod prehatchlings were sacrificed which do not fall under the above-mentioned directive and therefore the ethics approval is deemed unnecessary according to national and EU regulations. In addition, sampling of adult octopuses for this study originates from animals caught by artisanal fishermen for human consumption. This study was approved by an institutional review board, i.e. the institutional Ethic Committee, Órgano Encargado del Bienestar Animal del IIM-CSIC (OEBA-IIM; ES360570202001/17/EDUCFORM 07/CGM01). We confirm that our study is reported in accordance with the ARRIVE guidelines (https://arriveguidlines.org).

### Transcriptome sequencing and assembly

Two samples of mRNA of *O. vulgaris* were send to the Vienna Biocenter Facility (VBCF) for library construction and sequencing. Sample “Ovu1” included almost hatched individuals (stage XX), while “Ovu2” is a pooled sample of stages VIII, XI, XV, XVIII. RNA-seq libraries were constructed with a Lexogen SENSE mRNA-Seq Library Prep Kit V2 and sequenced with an Illumina Hi-Seq 2500 generating paired-end, stranded 125 bp libraries resulting in 53,523,481 (ovu1) and 63,328,653 (ovu2) paired end reads. The overall transcriptome assembly follows the procedure performed by De Oliveira et al.^42^. The short‐read libraries were preprocessed using Trimmomatic v. 0.36^43^ to remove known specific Illumina adapters from the paired‐end libraries (Illumina universal adapter). Filtering by quality and length was performed with a SLIDINGWINDOW:4:15 MINLEN:36. First and last nucleotides from reads with low quality score were clipped and the library file was converted into FASTA format using fq2fa from SeqKit version 0.11.0^44^. Quality of the initial and filtered library was assessed with the software FastQC v.0.11.8^45^ considering quality score of the bases, GC‐content, and read the best fit amino acid substitution length. 13.33% (ovu1) and 16.23% (ovu2) of reads were excluded during the preprocessing procedure resulting in a total of 46,386,109 (ovu1) and 53,052,713 (ovu2) reads. The assemblies and all downstream analyses were conducted with a high‐quality and clean library. The filtered transcriptome was assembled into contiguous cDNA sequences with IDBA_tran v1.1.3 software^46^ using the default settings (except: − mink20 − maxk 80 − step5). The resulting assembly was assessed using the tool QUAST (available at: http://quast.bioinf.spbau.ru)^47^. The number of contigs was 16,723 sequences (ovu1) and 18,551 (ovu2). Raw reads obtained by Illumina sequencing as well as the assembled transcriptomes are accessible on Zenodo (https://doi.org/10.5281/zenodo.8136693).

### Orthology analysis pipeline

Amino acid sequences of putative *Hox* and *ParaHox* genes of *O. vulgaris* were aligned together with bilaterian *Hox* and *ParaHox* amino acid sequences retrieved from the NCBI gene database (Supplementary Tables 1, 2), using ClustalOmega^48^ from webservice and then trimmed using ClipKit v1.4.0^49^. The best fit amino acid substitution model was obtained using ProtTest3^50^ using the AIC criterion. Afterwards, to assess the orthology relationships, a maximum likelihood analysis was performed using MrBayes^51^, for 14 million of generations, sampling every 1000 generations with eight chains and burn-in of 25% of trees. Later, the consensus tree was visualized using FigTree v1.4.4^52^.

### Probe design (PCR/sequences/transcriptome-screening)

Gene orthologs were identified in both transcriptomes using BLAST+^53^ sequence alignment. The accession numbers are stated in the supplementary material. DIG-labeled RNA probes were synthesized by in vitro transcription using the amplicons obtained by PCR from the cDNA of *O. vulgaris.* The primer sequences used for the amplification are mentioned in Supplementary Table 3.

### In-situ hybridization experiments

Whole-mount *in-situ* hybridization experiments were carried out as described previously by Wollesen et al.^34^. In brief, stage XIV (mid-embryogenesis) and stage XVIII (late-embryogenesis) individuals were rehydrated through a series of methanol and PBST buffer (PBS, pH = 7.4, with 0.1% Tween 20). Then incubated in proteinase K for 6 min at 37 °C, the reaction was stopped washing twice the samples with ice-cold PBST. To reduce unspecific probe binding the embryos were transferred to PBST with 1% triethanolamine and 0.3% acetic anhydride. Then, the embryos were fixed with 4% paraformaldehyde for 1 h and washed with PBST. Afterwards, the embryos were permeabilized using prehybridization buffer (50% formamide, 5× SSC, 100 µg/mL heparin, 5 mM EDTA, 100 µg/mL yeast tRNA, 0.1% Tween 20, 5% dextran sulfate) over night at 63 °C. The DIG-Labeled probes were denatured at 85 °C for 10 min, then resuspended at a 2 µL/mL concentration in the prehybridization buffer. Afterwards, embryos were incubated into this solution over night at 63 °C. Next, the samples were washed in sequentially decreasing concentrations from 4x saline-sodium citrate (SSC) buffer to 1x SSC at the hybridization temperature. Subsequently, after washing with PBST the samples were treated with MAB buffer (0.1 M maleic acid, 0.15 M sodium chloride and 0.1% Tween 20).

Afterwards, the embryos were incubated in blocking solution [MAB buffer with 10% Blocking reagent (Roche)] for 3 h at room temperature. Then, the samples were transferred to blocking solution with 1:2500 Anti-Digoxigenin- AP Fab fragments (Roche) overnight at 4 °C to detect the DIG label. The excess of antibody was removed using PBST. The samples were equilibrated in 100 mM NaCl, 50 mM ${\mathrm{MgCl}}_{2}$ and 0.1% Tween 20. To visualize the expression pattern in the embryos the samples were incubated for approximately 1 h at room temperature in color reaction buffer (4.5 µL/mL NBT, 3.5 µL/mL BCIP and 7.5% polyvinyl alcohol). The reaction was stopped and the samples were postfixed in 4% paraformaldehyde.

### Microscopy

The samples were mounted in 2,2′-thiodiethanol (TDE; Sigma-Aldrich) to visualize the expression patterns using an Olympus BX53 Upright Microscope.

## Results

### Gene orthology analysis

The trimming of the sequences retrieved by Clipkit excluded 240 sites (13.26%) of the total alignment. After manual checking of the alignment, best fit model assessment by Prottest selected the VT + G + F + I to be the best approximating model with an AIC score of 1. After including this model in the parameters of MrBayes, convergence (< 0.01) was achieved after 9 million generations. All studied *Hox* and *ParaHox* genes of *Octopus vulgaris* clustered with their respective bilaterians orthologs (Supplementary Fig. 1). Sequences of putative *Hox1, Hox5, * and *Xlox* cluster close to orthologs of *Euprymna scolopes.* For *Hox3, Lox4*, and *Gsx* the closest orthologs correspond to the ones of the cephalopods *Xipholeptos notoides* (former taxonomic name: *Idiosepius notoides*) and *E. scolopes* (Supplementary Fig. 1). According to our orthology analysis the identity of *Octopus vulgaris* *Hox1, Hox3, Hox5, Lox4, Lox2, Gsx,* and *Xlox* has been corroborated.

### *Hox* gene expression

#### *Hox1*-expression during stage XIV

**Figure 2:**
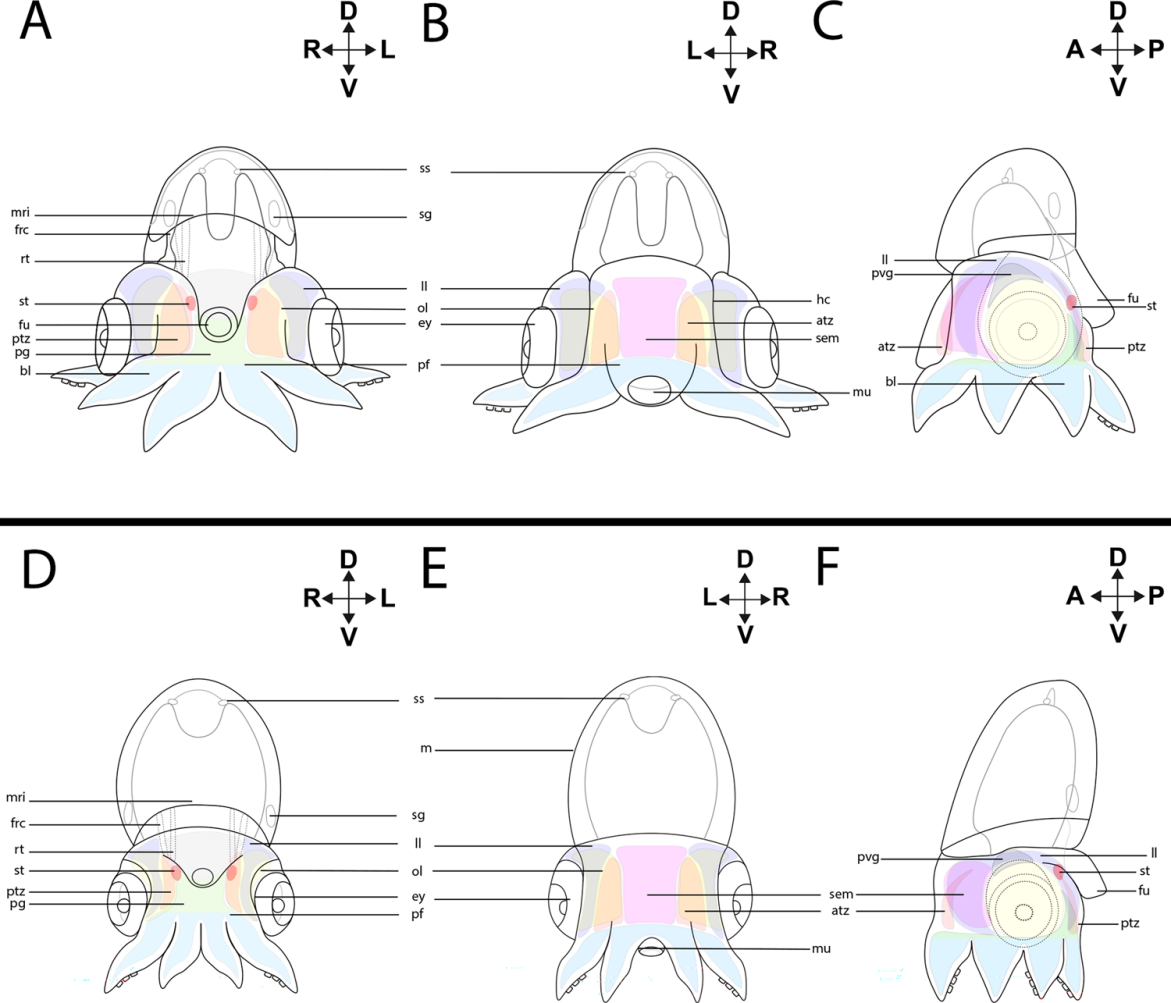
Topology of stage XIV and XVIII embryos of *Octopus vulgaris*. Dorsal (D)–ventral (V), anterior (A)–posterior (P), and left (L)–right (R) axes indicate the orientation. Lateral views (**C**,**F**). Anterior views (**B**,**E**) and posterior views (**A**,**D**). Stage XIV (**A**–**C**), stage XVIII (**D**–**F**). (**A**) For stage XIV, the shell sac (ss) is present in the dorsal region of the mantle (m), ventrally there are the stellate ganglia (sg), and the mantle rim (mri) delimits the border of the mantle. Associated with the funnel tube there are two muscles, the funnel retractor (frc) and the *rect.* *abdominalis* (rt)*.* Anterior to the funnel tube (fu), there are the statocysts (st) (red). In the head region, on the posterior side structures related to the central nervous system can be observed. Next to the eye (ey), and covering the optic lobes (ol) (yellow) the lateral lips (ll) (purple) are located, while the posterior transition zone (ptz) is labeled in orange. In the ventral region, the pedal ganglion (pg) (green) is connected to the the brachial lobes (bl) (blue) through the arm pillars (pf). (**B**) From the anterior view, the supraesophageal mass (sem) is located in between the optic lobes and the anterior transition zone (atz) (orange). From this perspective the mouth (mu) and the head cover (hc) are visible. (**C**) From the lateral side, the palliovisceral ganglion (pvg) (black) on top of the pedal ganglion is visible. (**D–F**) For stage XVIII the distribution of internal structures follows the previous description.

**Figure 3:**
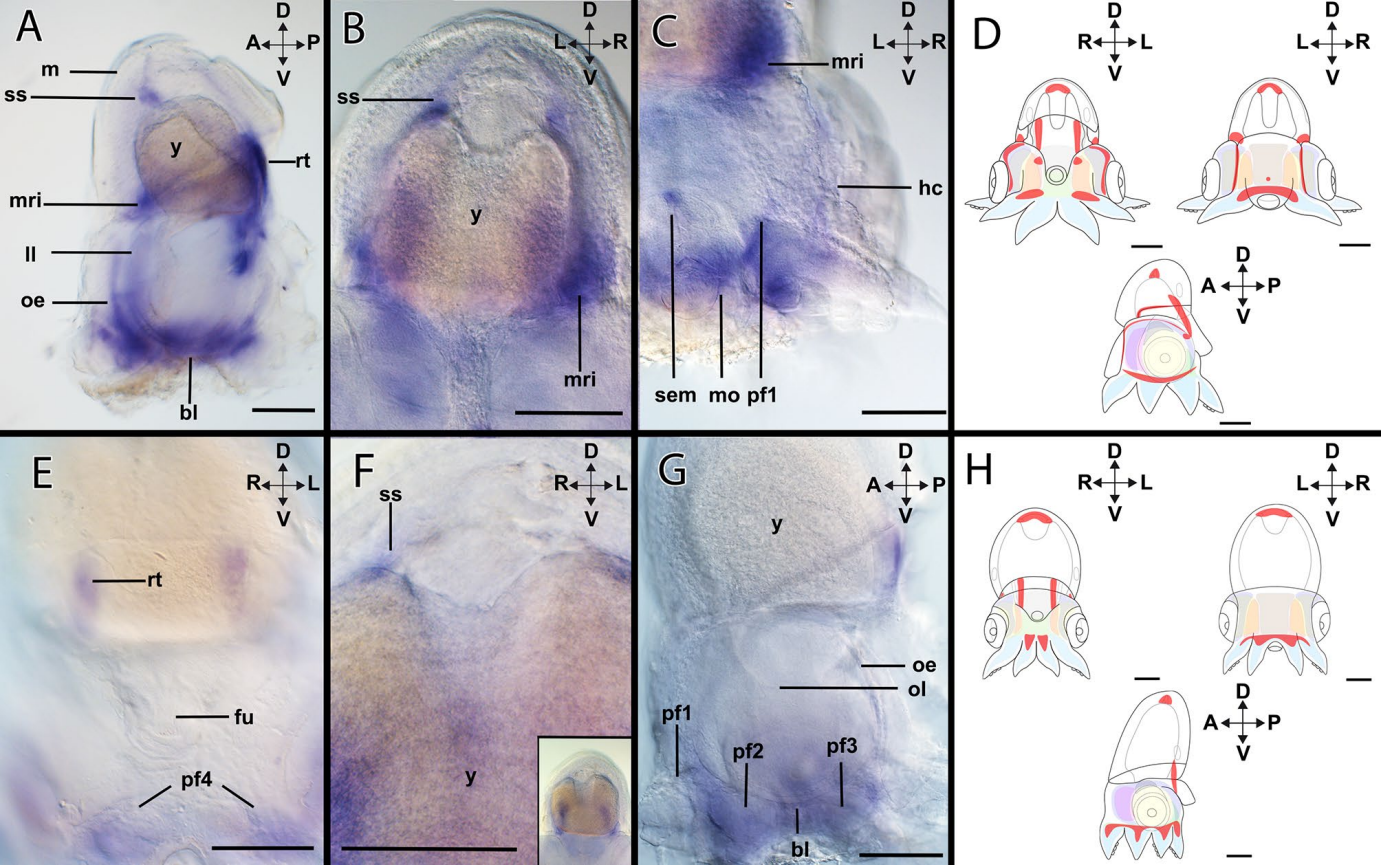
Expression of *Hox1* in developmental stages of *Octopus vulgaris.* Dorsal (D)–ventral (V), anterior (A)–posterior (P), and left (L)–right (R) axes indicate the orientation. Lateral views (**A**,**F**). Anterior view (**B**,**C**) and (**D**,**E**) (posterior view). Stage XIV (**A**–**C**), stage XVIII (**D**–**F**). (**A**) In stage XIV individuals, *Hox1* transcripts are present in the lateral lips (ll), the ventral ocular edges (oe), and the brachial lobes (bl). In the mantle (m) region, there is expression in the mantle rim (mri), and the retractor muscle (rt). (**B**) Expression of *Hox1* in the shell sac. (**C**) In the anterior region, *Hox1* is weakly expressed in the head cover (hc). In addition, there is an expression domain anterior to the suprasophageal mass (sem). In the arms, the expression of *Hox1* is clustered in the pillars of the arm pair (pf1). (**D**) Overview of the expression pattern of *Hox1* (red) in the embryo during stage XIV. (**E**) Stage XVIII, *Hox1*-expression is present in the retractor and on the arm pillars of the posterior arm pairs IV (pf4). (**F**) Expression is also visible in the shell sac in the mantle. (**G**) Expression domains of *Hox1* also comprise the brachial lobes ventrally to the optic lobes, connecting the arm pillars I and III (pf1, pf3). (**H**) Overview of the expression pattern of *Hox1* (red) in the embryo during stage XVIII. *fu* funnel, *ss* shell sac, *ol* optic lobe, *y* yolk. Scale bars: 200 µm.

*Hox1*-expression (Figs. 2A–C, 3A–D) is present in the shell sac (Fig. 3A,B) and the retractor muscle *rect. abdominalis* (Fig. 3A; Supplementary Fig. 2A). On the ventral side of the embryo, *Hox1* + cells are present in the brachial lobes (Fig. 3A). In the anterior head region, *Hox1* expression is associated with the dorsal region of the lateral lips and the ocular edges (Fig. 3A). In the mantle region, *Hox1* is restricted to the mantle rim (Fig. 3A,B). On the posterior side, *Hox1* is expressed in the funnel pouches (Supplementary Fig. 2A), while expression is also present close to the supraesophageal mass (Fig. 3C). In addition, *Hox1* transcripts are present in the pillar of arm pair I (Fig. 3C and Supplementary Fig. 2B). Additionally, the region limiting the head cover expresses *Hox1* (Fig. 3C).

#### *Hox1*-expression during stage XVIII

*Hox1* expression domains (Figs. 2D–F, 3E–H) include the retractor muscle *rect. abdominalis*, the pillars of the arm pair IV (Fig. 3E), and the funnel pouches (Supplementary Fig. 2C). In the dorsal mantle region, *Hox1*-expression is visible in the shell sac (Fig. 3F). In the head region, *Hox1*-expression is present ventrally to the optic lobes, in the brachial lobes, and the pillars of arm pairs II and III (Fig. 3G). Moreover, there is *Hox1*-expression delimitating the ocular edges (Supplementary Fig. 2D). All four arm pillars (I–IV) exhibit *Hox1*-expression that is continuous to the *Hox1*-expression of the brachial lobes (Fig. 3G).

#### *Hox3*-expression during stage XIV

**Figure 4:**
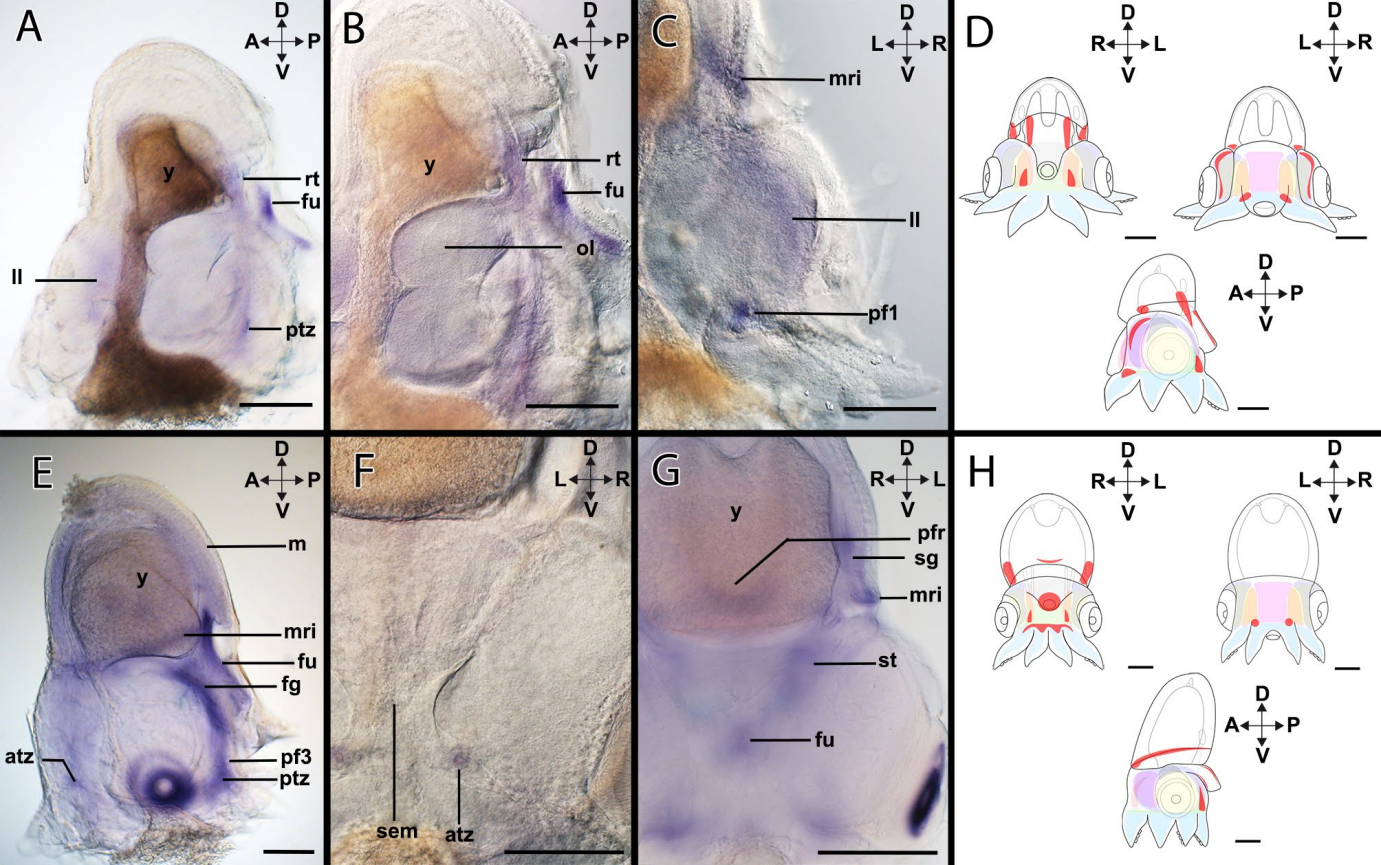
Expression of *Hox3* in developmental stages of *Octopus vulgaris.* Dorsal (D)–ventral (V), anterior (A)–posterior (P), and left (L)–right (R) axes indicate the orientation. Lateral views (**A**,**B**,**D**); anterior views (**C**,**E**); posterior view (**F**). Stage XIV (**A**–**C**), Stage XVIII (**D**–**F**). (**A**) Stage XIV specimens show *Hox3*-expression in the posterior region around the posterior transition zone (ptz), in the anterior region of the lateral lips (ll), in the funnel (fu), and in the retractor (rt). (**B**) *Hox3* is expressed in the funnel. (**C**) *Hox3* transcripts are located in the lateral lips and in a defined cluster in arm pillar I (pf1). In addition, expression is also present in the mantle rim (mr). (**D**) Overview of the expression pattern of *Hox3* (red) in the embryo during stage XIV. (**E**) In stage XVIII, *Hox3*-expression is located in the posterior region of the embryo. The mantle rim, the funnel, and the funnel gland (fg) exhibit *Hox3* transcripts. On the ventral side, *Hox3* expression domains are located in the anterior transition zone (atz), the posterior transition zone, and in the pillar of the arm pair III (pf3). (**F**) *Hox3*-expression is observed in two symmetrical dots in the anterior transition zone anterior to the supraesophageal mass (sem). (**G**) In the posterior region, *Hox3*-expression is present in the posterior funnel rim (pfr), the stellate ganglia (sg), and the mantle rim. (**H**) Overview of the expression pattern of *Hox3* (red) in the embryo during stage XVIII. *st* statocysts, *ol* optic lobe, *y* yolk. Scale bars: 200 µm.

*Hox3*-expression (Figs. 2A–C, 4A–D) is present in the anterior region of the lateral lips and the posterior transition zone (Fig. 4A). *Hox3* is only expressed in the anterior region of the funnel (Fig. 4A,B) and in the region of the retractor muscle *rect. abdominalis* (Fig. 4B). In the anterior region, *Hox3* is expressed in the lateral lips near the limit of the head cover (Fig. 4C). In the mantle, there is additional expression localized in the mantle rim (Fig. 4C) and the funnel pouches (Supplementary Fig. 3A). In the pillars of arm pair I (Supplementary Fig. 3B), *Hox3* transcripts form two equidistant expression domains (Fig. 4C).

#### *Hox3*-expression during stage XVIII

*Hox3*-expression (Figs. 2D–F, 4E–H) is predominantly posterior, being present in the funnel tube, the posterior transition zone, and in the pillars or the arm pairs III and IV (Fig. 4E; Supplementary Fig. 3C). In the funnel tube, the expression of *Hox3* involves the inner cells of the tube and covers the funnel gland (Fig. 4E). However, the expression is not present in the funnel rim (Fig. 4E). In the anterior region, the expression of *Hox3* is localized in the seam between the head cover and the mantle, an expression domain that is extended through the mantle rim (Fig. 4E). *Hox3* is expressed in two domains in the region of the anterior transition zone close to the supraesophageal mass (Fig. 4F) and in the stellate ganglia and the posterior funnel rim (Fig. 4G).

#### *Hox5*-expression during stage XIV

**Figure 5:**
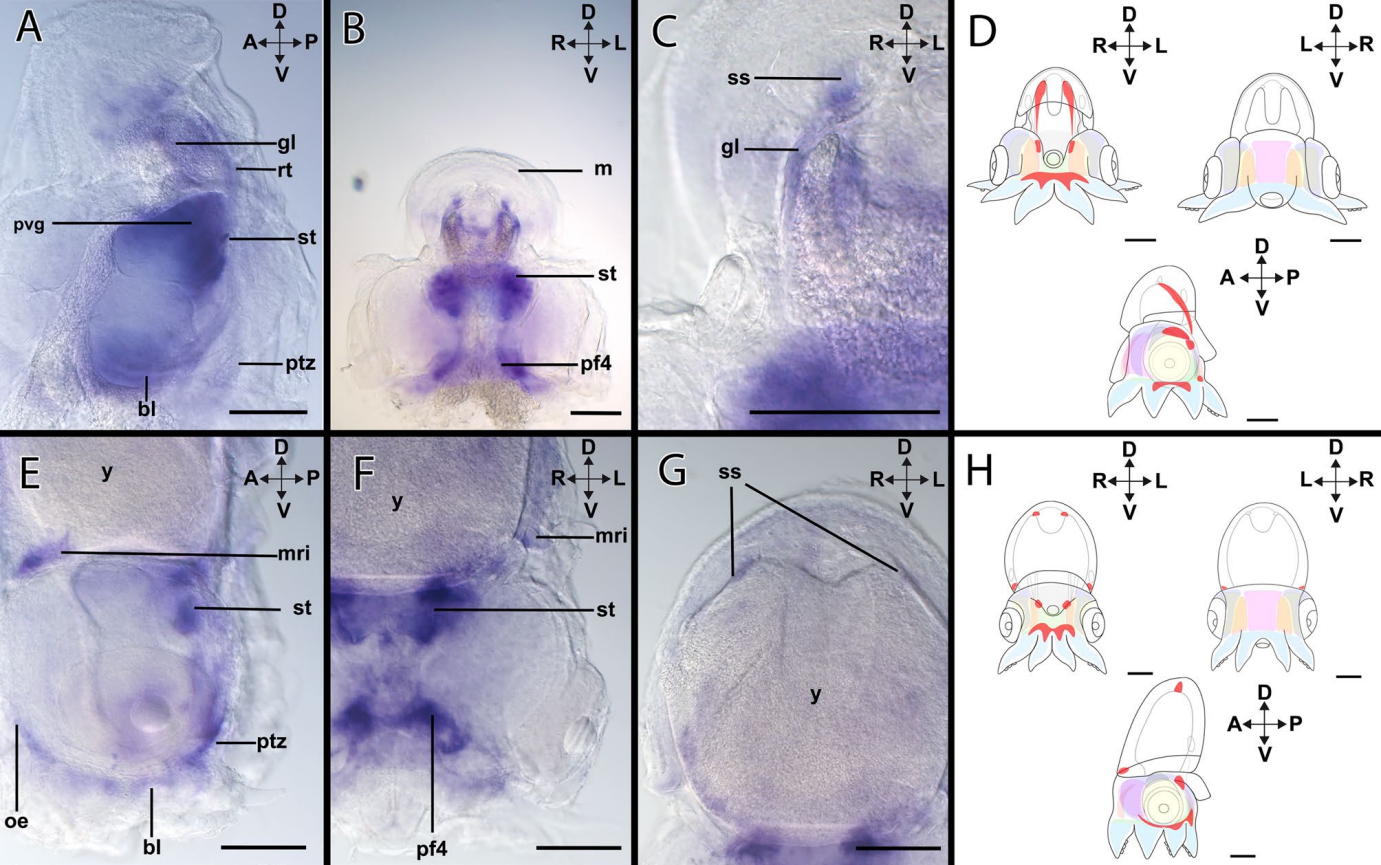
Expression of *Hox5* in developmental stages of *Octopus vulgaris.* Dorsal (D)–ventral (V), anterior (A)–posterior (P), and left (L)–right (R) axes indicate the orientation. All posterior views (**B**,**C**,**E**,**F**) with exception of (**A**,**D**) lateral views. Stage XIV (**A**–**C**), Stage XVIII (**D**–**F**). (**A**) In the mantle, the *Hox5* is expressed in the gill lamellae (gl), and the retractor (rt). In the head region, the expression of *Hox5* is located in palliovisceral ganglion (pvg) behind the statocysts (st). Moreover, there is expression in brachial lobe and in the posterior transition zone (ptz). (**B**) From the posterior view, there is evidence of *Hox5* transcripts in the arm pillars III and IV. (**C**) In the mantle region, *Hox5* expression can be found in the gill lamella and the shell sac (ss). (**D**) Overview of the expression pattern of *Hox5* (red) in the embryo during stage XIV. (**E**) In stage XVIII individuals, expression is observed in the mantle rim (mri). In the head region, the expression of *Hox5* is present along the ocular edges (oe), the brachial lobes, and the posterior transition zone. (**F**) *Hox5*-expression is visible in the arm pillars III and IV. (**G**) Close-up of the mantle region with *Hox5* expression in the shell sac. (**H**) Overview of the expression pattern of *Hox5* (red) in the embryo during stage XVIII. *bl* brachial lobe, *m* mantle, *st* statocysts, *y* yolk. Scale bars: 200 µm.

*Hox5*-expression (Figs. 2A–C, 5A–D) is mostly located in the posterior region of the embryo (Fig. 5A). In the head region close to the ventral side, expression is located in the brachial lobes and the posterior transition zone (Fig. 5A). In the dorsal head region, expression is located in the palliovisceral ganglion, anterior to the statocysts (Fig. 5A). On the posterior side, *Hox5* transcripts are present in the pillars of the arm pairs III and IV (Fig. 5B). In addition, *Hox5-*expression is present in the region of the gill lamellae and the shell sac (Fig. 5C).

#### *Hox5*-expression during stage XVIII

There is expression of *Hox5* (Figs. 2D–F, 5E–H) in the mantle and in the dorsal mantle rim (Fig. 5E,F). *Hox5*-expression is present along the ocular edges, in the brachial lobes, and the posterior transition zone (Fig. 5E). Additional expression is present in the pillars of the arm pair III and IV (Fig. 5F) and in the region of the shell sac (Fig. 5G).

#### *Lox4*-expression during stage XIV

**Figure 6:**
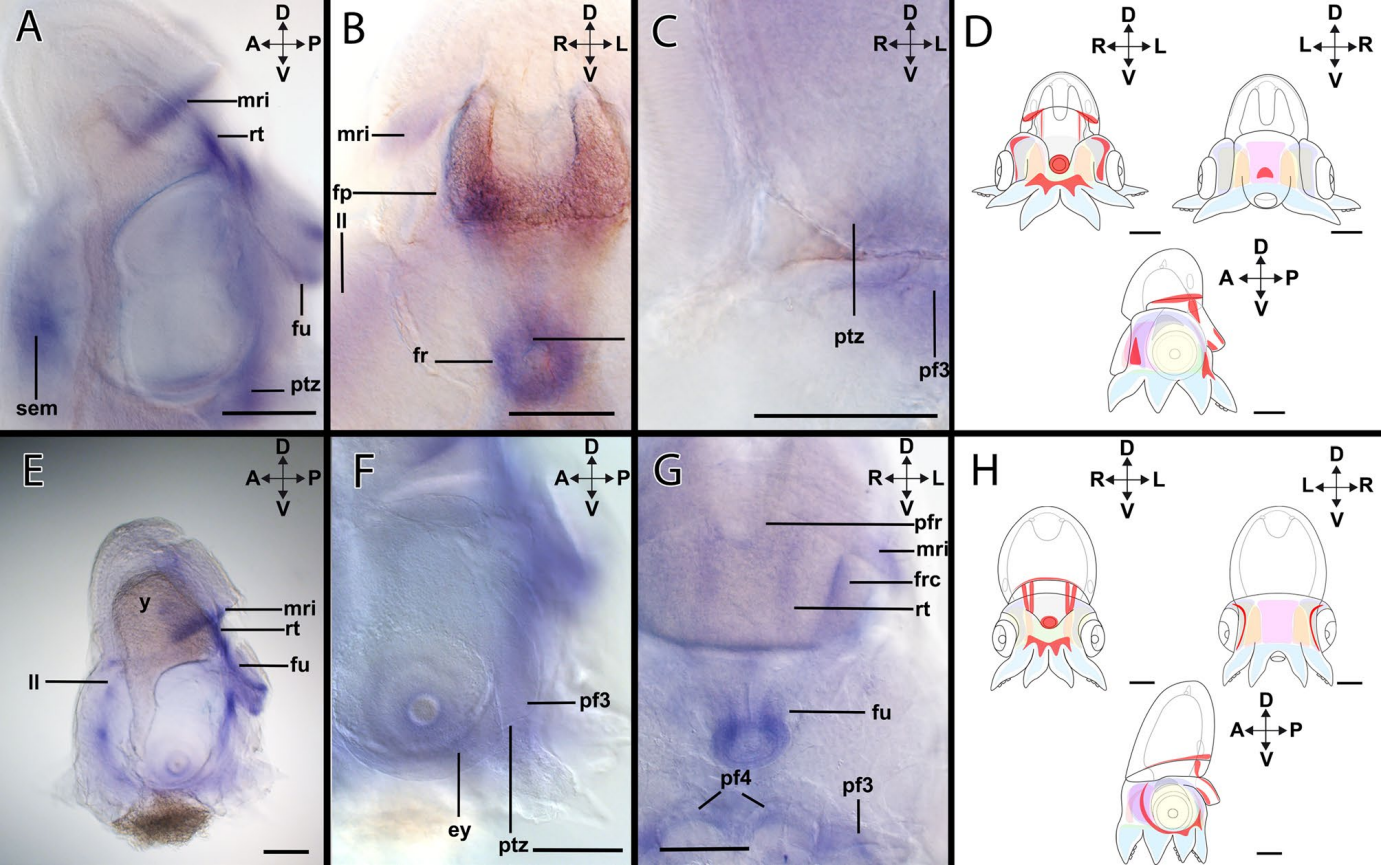
Expression of *Lox4* in developmental stages of *Octopus vulgaris.* Dorsal (D)–ventral (V), anterior (A)–posterior (P), and left (L)–right (R) axes indicate the orientation. Posterior views (**B**,**C**,**F**) and lateral views (**A**,**D**,**E**). Stage XIV (**A**–**C**), stage XVIII (**D**–**F**). (**A**) In stage XIV individuals, *Lox4* is expressed in the mantle rim (mri) and in the retractor (rt), as well as in the funnel (fu). In the ventral region, *Lox4* transcripts are accumulated in the posterior transition zone (ptz) and in the supraesophageal mass (sem). (**B**) On the posterior side, *Lox4* is expressed around the funnel rim (fr) and in the dorsal region of the lateral lips (ll). In the mantle region, *Lox4*-expression is located around the funnel pouches (fp). (**C**) In the ventral region, *Lox4*-expression is located in the arm pillar III (pf3) and the posterior transition zone. (**D**) Overview of the expression pattern of *Lox4* (red) in the embryo during stage XIV. (**E**) In XVIII individuals, *Lox4*-expression is present in the mantle rim, the retractor, the anterior region of the lateral lips. (**F**) In the ventral region, close to the eye (ey), *Lox4* is expressed in the arm pillar III and the posterior transition zone. (**G**) In the posterior region, expression of *Lox4* is restricted to the posterior funnel rim, the mantle rim, the retractor, the funnel retractor and the funnel tube. In addition, there is expression in the pillars of the arm pairs III and IV. (**H**) Overview of the expression pattern of *Hox5* (red) in the embryo during stage XVIII. *frc* funnel retractor, *y* yolk. Scale bars: 200 µm.

In stage XIV individuals, expression of *Lox4* (Figs. 2A–D, 6A–D) can be found anteriorly in the buccal area, near the supraesophageal mass (Fig. 6A). In the mantle region, *Lox4* expression is present in the *rect. abdominalis* and extends towards the funnel tube with strong expression near the funnel rim (Fig. 6A,B). In the mantle rim, the *Lox4*-expression pattern is more defined in the posterior region (Fig. 6A). In the head region of the embryo, *Lox4*-transcripts are present in the posterior transition zone (Fig. 6A,C) and in the arm pillars of the arm pairs III and IV (Fig. 6C, Supplementary Fig. 4A). In addition, *Lox4* + cells are present on the dorsal side of the lateral lips and the funnel pouches (Fig. 6B).

#### *Lox4*-expression during stage XVIII

*Lox4*-expression (Figs. 2D–F, 6E–H) is present in the mantle rim and extends to the seam between the head cover and the mantle, but the expression is stronger in the posterior region of the embryo (Fig. 6E). On the anterior side of the head, *Lox4* is present in the lateral lips (Fig. 6E). On the posterior side, *Lox4* is expressed in the muscle *rect. abdominalis *(Fig. 6E). From this muscle, expression extends towards the funnel tube, covering the funnel gland and finishing in the funnel rim (Fig. 6E,G). On the ventral side of the head, the expression is localized in the posterior transition zone, the pillars, and the basal surfaces of arm pairs III and IV (Fig. 6F,G). Additionally, *Lox4* is expressed in the retractor muscle, the funnel retractor muscle, and the posterior funnel rim (Fig. 6G).

#### *Lox2*-expression during stage XIV

**Figure 7:**
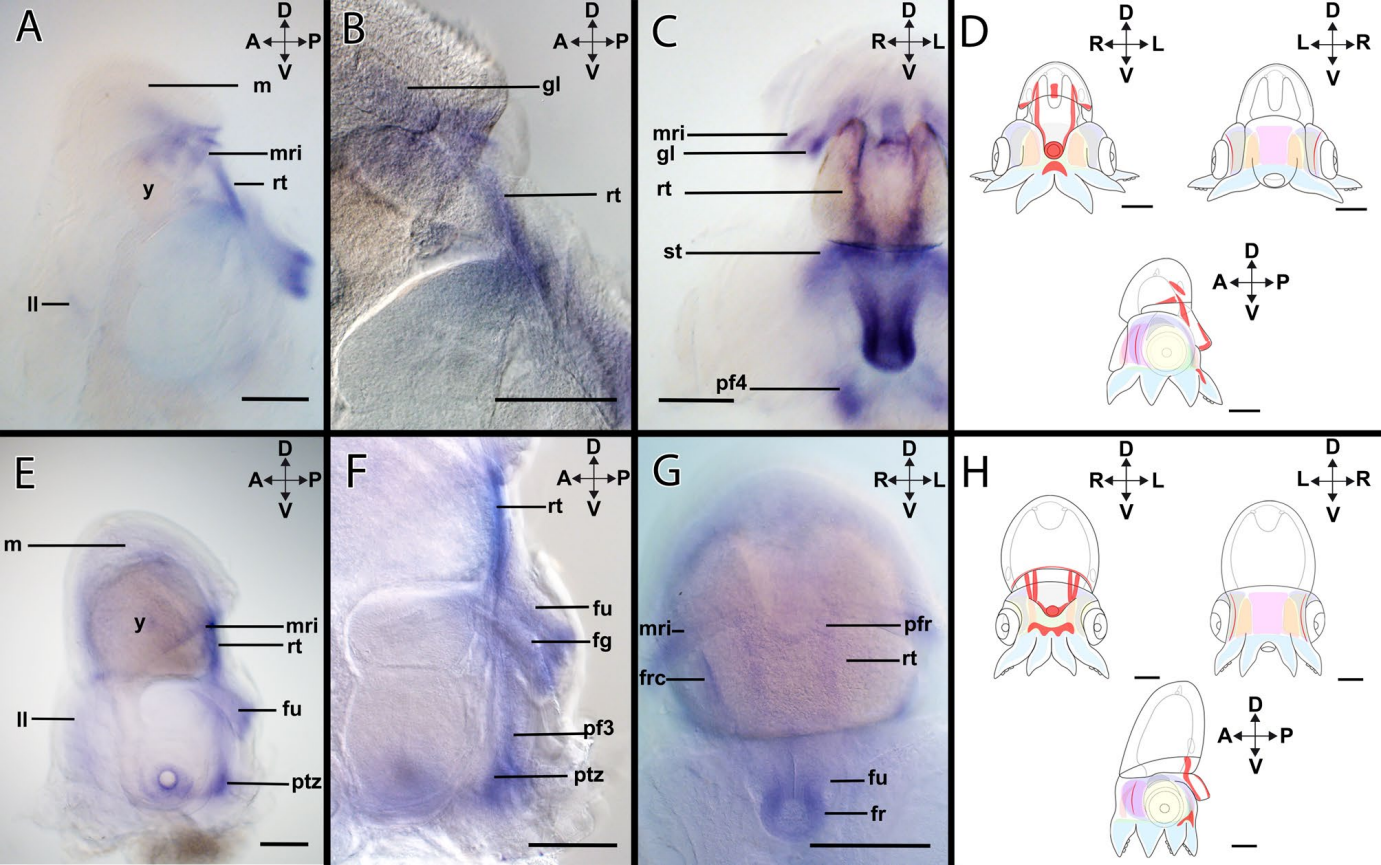
Expression of *Lox2* in developmental stages of *Octopus vulgaris.* Dorsal (D)–ventral (V), anterior (A)–posterior (P), and left (L)–right (R) axes indicate the orientation. All lateral views (**A**,**B**,**D**,**E**) with exception of (**C**) and (**F**) (posterior views). Stage XIV (**A**–**C**), stage XVIII (**D**–**F**). (A) In stage XIV, *Lox2* is expressed faintly in the anterior region of the lateral lips (ll). Strong *Lox2-*expression was observed in the posterior region including the retractor muscle (rt) and the mantle rim (mri). (**B**) *Lox2*–expression is present in the retractor muscle. (**C**) In the posterior region, *Lox2*-expression is located in in the mantle rim, the gill lamellae (gl), the retractor muscle, the funnel rim, and the arm pillars III and IV (pf4). (**D**) Overview of the expression pattern of *Lox2* (red) in the embryo during stage XIV. (**E**) In stage XVIII, *Lox2*-expression domains are located in the posterior region of the embryo, but there is faint expression in the lateral lips. (**F**) In the posterior region, the expression of *Lox2* extends from the retractor muscles towards the funnel tube and ends in the ventral side close to the posterior transition zone, being also present in the pillars of the arm pair III. (**G**) On the posterior side, *Lox2* is expressed in the posterior funnel rim, the funnel retractor (frc), and the mantle rim. (**H**) Overview of the expression pattern of *Lox2* (red) in the embryo during stage XVIII. *fg* funnel gland, *fu* funnel, *gl* gill lamellae, *ll* lateral lips, *pf3* arm pillar 3, *ptz* posterior transition zone, *st* statocyst, *y* yolk. Scale bars: 200 µm.

Stage XIV individuals express *Lox2* (Figs. 2A–D, 7A–D) faintly in the lateral lips in the anterior region lips (Fig. 7A). *Lox2* is also expressed in the muscle *rect. abdominalis* and in the rim of the funnel tube (Fig. 7A,C). In the mantle, expression of *Lox2* is located in the gill lamellae and the mantle rim (Fig. 7B,C). Ventrally, the expression domain of *Lox2* is related to the arm pillars of the arm pair IV (Fig. 7C).

#### *Lox2*-expression during stage XVIII

The *Lox2-*expression pattern of (Figs. 2D–F, 7E–H) stage XVIII individuals resembles the one described for stage XIV individuals (Fig. 7E). In the anterior region, there is faint expression around the lateral lips (Fig. 7E), and the supraesophageal mass (Supplementary Fig. 5A). In the posterior region, *Lox2*-expression is present in the muscle *rect. abdominalis* near the mantle region (Fig. 7E). Ventrally, *Lox2* expression is restricted to the posterior transition zone and the pillars of the arm pairs III and IV (Fig. 7F, Supplementary Fig. 5B). In the funnel, *Lox2*-expression is located in the funnel gland and it extends until the rim (Fig. 7F,G). Posteriorly, *Lox2* is expressed in the funnel retractor and in the posterior funnel rim (Fig. 7G).

### *ParaHox* gene expression

#### *Gsx*-expression during stage XIV

**Figure 8:**
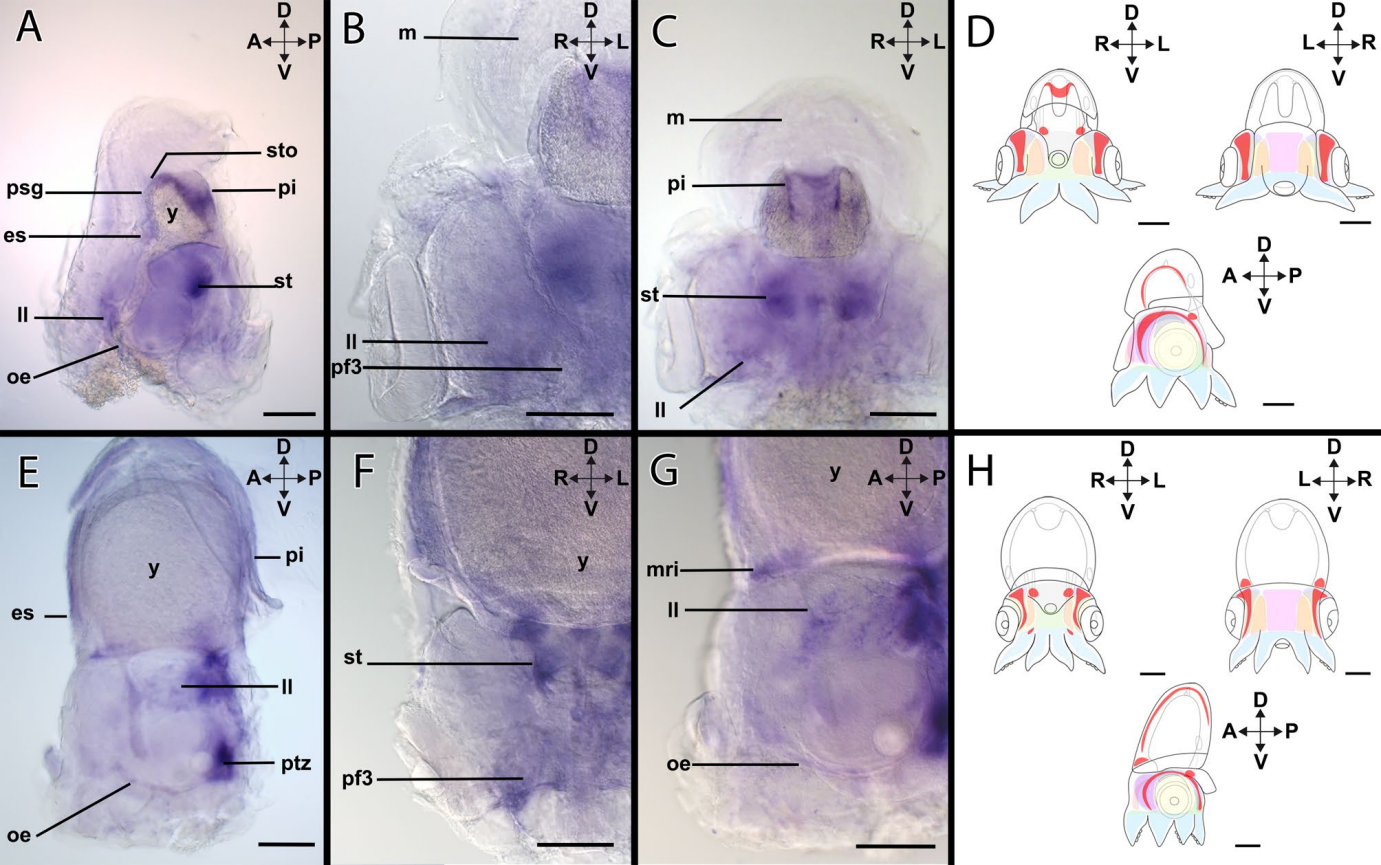
Expression of *Gsx* in developmental stages of *Octopus vulgaris.* Dorsal (D)–ventral (V), anterior (A)–posterior (P), and left (L)–right (R) axes indicate the orientation. Lateral views (**A**,**D**,**F**) and posterior views (**B**,**C**,**E**). Stage XIV (**A**–**C**), stage XVIII (**D**–**F**). (**A**) For stage XIV, *Gsx* is expressed in the digestive system of the embryo including the primordial intestine (pi), the stomach (sto), the posterior salivary glands (psg), and the terminal region of the esophagus (es). (**B**,**C**) *Gsx* transcripts are found in the head region in the lateral lips. (**D**) Overview of the expression pattern of *Gsx* (red) in the embryo during stage XIV. (**E**) In stage XVIII, the expression pattern of *Gsx* surrounds the yolk delimitating the primordial intestine, the posterior salivary glands, and the esophagus. (**F**) *Gsx*-expression is present in the posterior arm pillar of arm pair III. (**G**) The anterior mantle rim (mri), the lateral lips and the ocular edges express *Gsx*. (**H**) Overview of the expression pattern of *Gsx* (red) in the embryo during stage XVIII. *ll* lateral lips, *m* mantle, *oe* ocular edge, *pf3* pillar of arm pair III, *ptz* posterior transition zone, *st* statocyst, *y* yolk. Scale bars: 200 µm.

*Gsx* is expressed in the developing digestive system (Figs. 2A–D, 8A–D), i.e. faint expression is visible around the esophagus and stronger expression in the mid- and hindgut including the posterior salivary glands (Fig. 8A). *Gsx*-expression is also visible in the region of the lateral lips (Fig. 8B,C).

#### *Gsx*-expression during stage XVIII

Stage XVIII individuals express *Gsx* (Figs. 2D–F, 8E–H) in the dorsal region of the lateral lips (Fig. 8E,G) and in the ventral ocular edge (Fig. 8E,G). In addition, *Gsx* transcripts are located in the region of the developing digestive system adjacent to the internal yolk (Fig. 8E). In the ventral region of the embryo, *Gsx* expression is present in the posterior transition zone and the pillars of the arm pair III (Fig. 8F). In the ventral region, close to the mouth faint expression is visible (Supplementary Fig. 6A).

#### *Xlox*-expression during stage XIV

**Figure 9:**
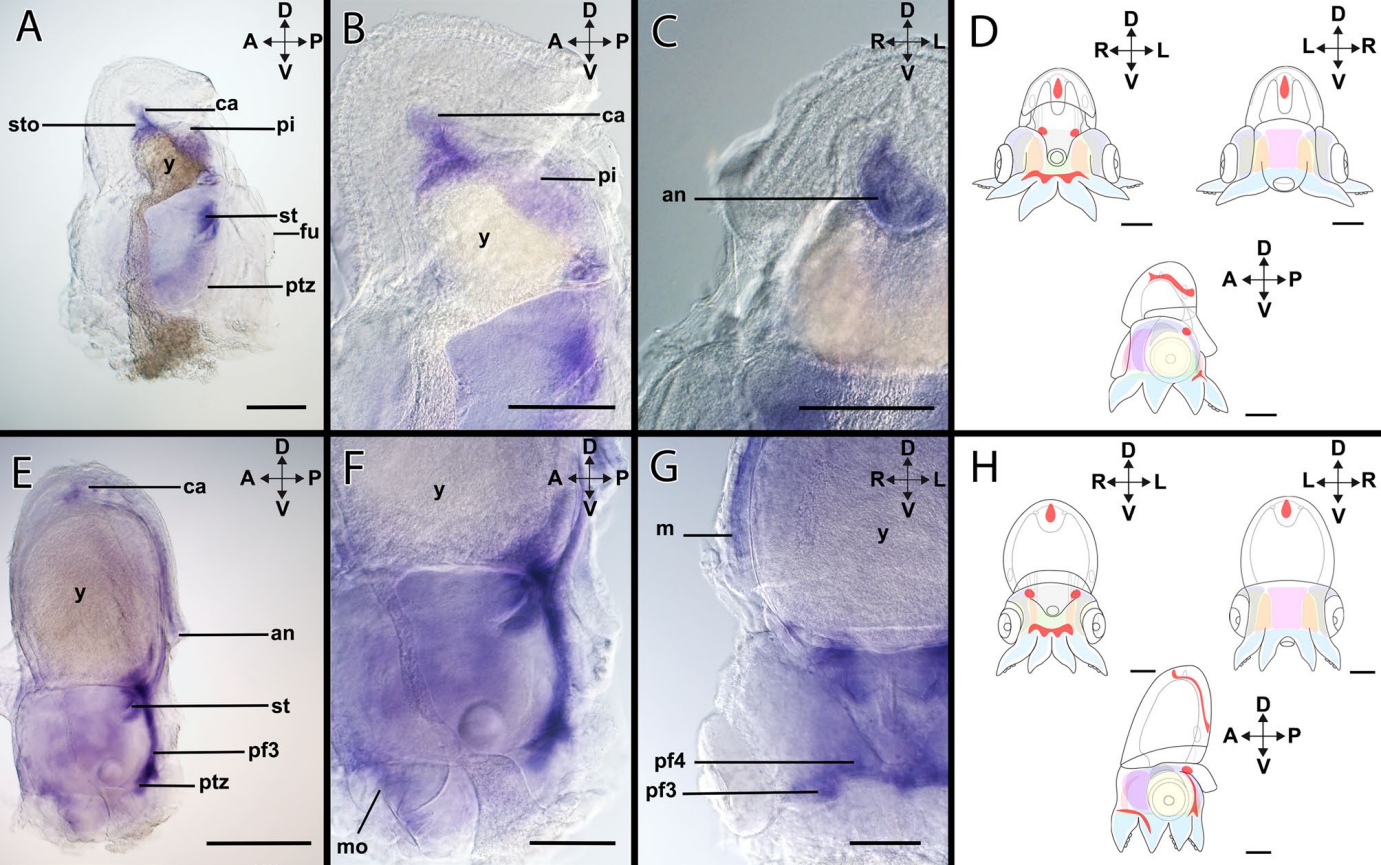
Expression of *Xlox* in developmental stages of *Octopus vulgaris.* Dorsal (D)–ventral (V), anterior (A)–posterior (P), and left (L)–right (R) axes indicate the orientation. All lateral views (**A**,**B**,**D**,**E**) with exception of (**C**) and (**F**) (posterior views). Stage XIV (**A**–**C**), stage XVIII (**D**–**F**). (**A**) In stage XIV individuals, *Xlox* expression is located in the region of the stomach (sto), the caecum (ca), and the primordial intestine (pi). (**B)** *Xlox* is expressed in the digestive tract close to the internal yolk. (**C**) The expression of *Xlox* is visible in region of the anus (an). (**D**) Overview of the expression pattern of *Xlox* (red) in the embryo during stage XIV. (**E**) In stage XVIII individuals, *Xlox* is expressed in the digestive tract between the anus and the caecum. The posterior transition zone in the head region expresses *Xlox* and the arm pillar III (pf3). (**F**) *Xlox* is expressed in the mouth (mo) region. (**G**) In addition, the arm pillars III and IV (pf4) express *Xlox*. (**H**) Overview of the expression pattern of *Xlox* (red) in the embryo during stage XVIII. *fu* funnel, *m* mantle, *ptz* posterior transition zone, *st* statocyst, *y* yolk. Scale bars: 200 µm.

*Xlox* is expressed in the developing digestive system in the region of the caecum, the hindgut, and the anus (Figs. 2A–D, 9A–D). In the head region, the *Xlox-*expression is present in the posterior transition zone (Fig. 9A). *Xlox* is also expressed in the arm pillars III and IV (Supplementary Fig. 7A).

#### *Xlox*-expression during stage XVIII

*Xlox-*expression during stage XVIII resembles the one of stage XIV (c.f. Fig. 9A–H). *Xlox* is expressed in the hindgut, i.e. in the region between the caecum and the anus (Figs. 2D–F, 9E). Further expression is present in the head region close to the supraesophageal region and the mouth (Fig. 9F, Supplementary Fig. 7B). In the posterior region, *Xlox* is expressed in the posterior transition zone (Fig. 9E,F) and the pillars of the arms lll and lV (Fig. 9G).

## Discussion

### Post-cerebral *Hox* expression

The present study in combination with a revised analysis of previous datasets shows that cephalopods exhibit more similarities on the gene expression level with other mollusks and bilaterians than previously anticipated. Similar to *Euprymna scolopes*, no *Hox* gene is expressed in the anterior-most brain region, i.e. the cerebral ganglia (prospective supraesophagael mass incl. optic lobes) of *Octopus vulgaris* ^13^, present study]. In contrast, *Otx* is expressed in this domain and *Pax2/5/8* is expressed in adjacent more posterior brain regions, i.e. the anterior basal lobes and the interbasal lobes of *X. notoides*^54,55^. *Gbx* is co-expressed with other *Hox* genes in posterior-most brain regions such as the posterior and middle subesophageal masses and stellate ganglia of *Sepia officinalis*^13,54,56^, (present study). This anterior–posterior sequence of expression is similar to the condition found in other bilaterians^57^. In *O. vulgaris*, all *Hox* genes are exclusively expressed posterior to the esophagus with exception of a small *Hox1* expression domain anterior to the esophagus in the developing supraesophageal mass (Fig. 3C). Expression of anterior *Hox* genes in the supraesophageal mass has only been described so far for *Hox3* in *S. officinalis*^54^.

### Remnants of staggered *Hox* gene expression in coleoid cephalopods

**Figure 10:**
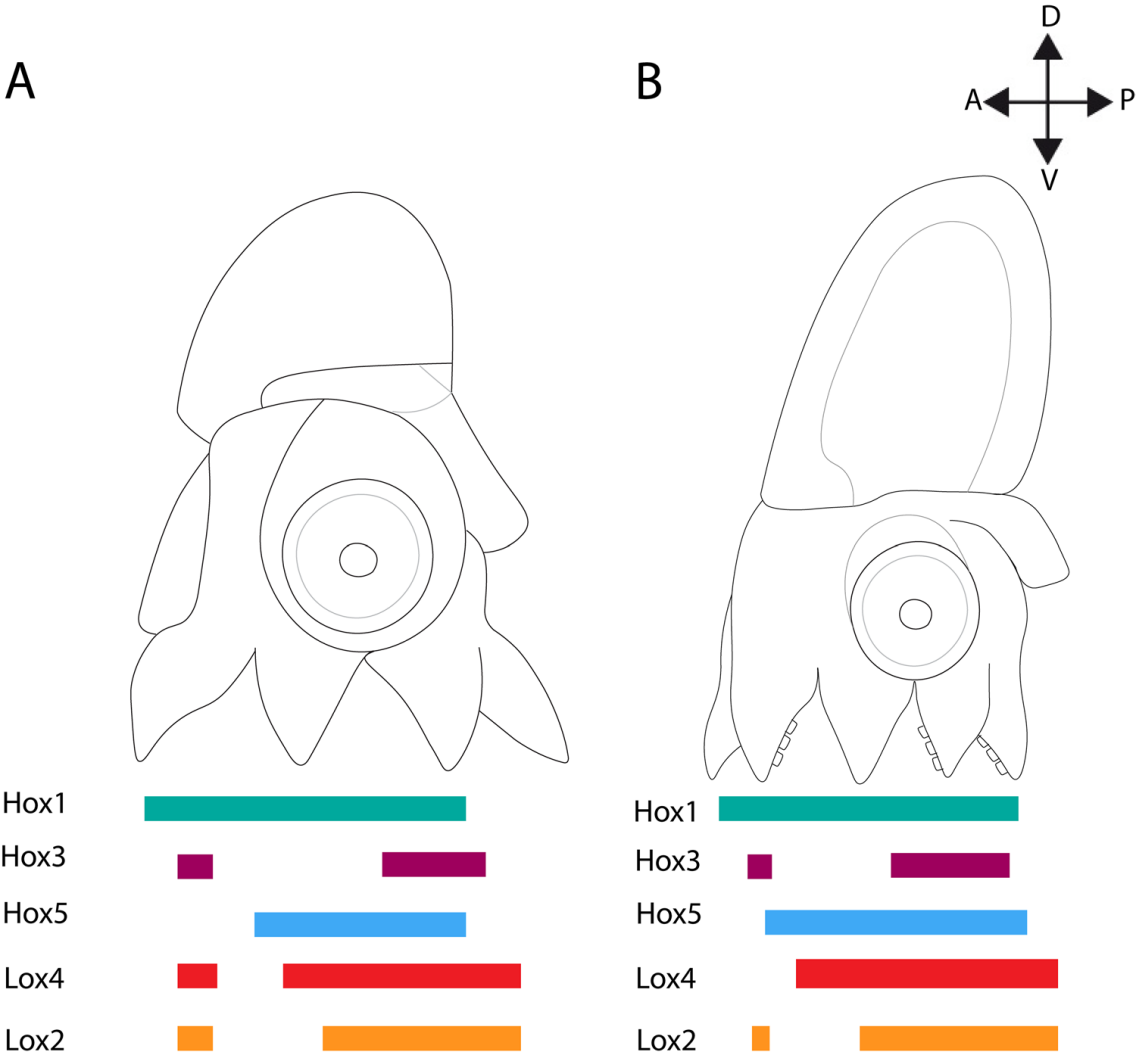
Near-to-staggered *Hox* genes expression in developmental stages of *Octopus vulgaris.* Dorsal (D)–ventral (V), anterior (A)–posterior (P) axes indicate the orientation. (**A**) Anterior (*Hox1, 3, 5*) and central (*Lox2*, *4*) *Hox* genes of stage XIV (**A**) and stage XVIII individuals (**B**) are expressed in a near-to-staggered fashion.

Based on a previous study on scaphopod mollusks it was assumed that the last common ancestor of cephalopods also exhibited staggered *Hox* expression^34^. While we were able to present *Lox2*-expression patterns for the first time for a cephalopod^3,13^, we were not able to study the expression of *Hox4*, *Lox5*, *Hox7*, *Post1*, and *Post2* since these were either not found in the transcriptomes or the published genome of *Octopus vulgaris* or templates for riboprobe syntheses could not been amplified by PCR. Our study shows that *Hox* genes are expressed in a near-to-staggered fashion in two developmental stages of *O. vulgaris* (Fig. 10). This is particularly obvious in stage XVIII with only *Lox2* violating staggered *Hox* expression (Fig. 10B). In stage XIV individuals, *Lox4* and *Lox2* violate staggered expression (Fig. 10A). Since *O. vulgaris*, *E. scolopes*, and other mollusks show traces of staggered *Hox* expression, this condition was probably already present in the last common ancestor of cephalopods and mollusks^26,34^ (Fig. 1F). Although it is unknown where *Hox* genes are expressed in nautiloid cephalopods, staggered expression and traces of staggered expression in coleoid cephalopods and their conchiferan relatives render it highly probable that also nautiloid embryos show staggered *Hox* expression. The presence of a full set of *Hox* genes including *Hox2* in *Nautilus* corroborates that the rather simple nautiloid body plan shares affinities with the one of the conchiferan relatives^58–60^.

### Are *Hox* genes only expressed in a staggered fashion or is their expression also evolutionarily conserved in homologous organs among cephalopods?

The common octopus *O. vulgaris* and the bobtail squid *E. scolopes* express given *Hox* genes in homologous organ systems as well as in a number of domains that are unique for both species (present study^13^). For example, *Hox1* is expressed in arm pairs I and II of both species (stages 19–27 in *E. scolopes*). *Hox3* is expressed in the region between arm pair I and II in both species and in the stellate ganglia. It is also expressed in the funnel of both species and the cuttlefish *Sepia officinalis*^54^. *Hox5* is expressed in the arm pairs III and IV and in the palliovisceral ganglia in both species. *Lox4* expression is located in the arm pair III and in the funnel of both species. In this sense, *Hox* expression is also staggered in these homologous body regions.

### Cephalopod plesiomorphic traits are corroborated by *Hox* gene expression

Although the cephalopod body plan deviates considerably from the one of other mollusks, a number of organs have molluscan homologs according to classical morphological and ontogenetic studies^59^. Among these organs are the mantle with a shell gland (cephalopod shell sac) and derivatives from the foot (cephalopod arms and funnel). For example, *Hox1* has been shown to be expressed in the shell glands of all mollusks except for aplacophorans and monoplacophorans which have not been studied yet^26^. For cephalopods, it has been hypothesized that *Hox1* expression has been lost due to shell reduction since no *Hox* gene has been documented to be expressed in the region of *E. scolopes*^13^. In *O. vulgaris*, we found *Hox1* to be expressed in the shell field of mid-stage embryos but not older embryos (c.f. Fig. 3B,F). It could well be that other—not documented—developmental stages express *Hox1* in the shell field of *E. scolopes*.

*Hox5* is expressed in the shell glands of stages XIV and XVIII in *O. vulgaris* as well as the mantle tissue of adult nautiluses (nautiloid embryonic condition unknown)^60^. Furthermore, the veliger larva of the gastropod *Gibbula varia* expresses *Hox5* in the mantle covering the visceral mass and digestive gland^31,32^. The vast majority of *Hox* genes are also expressed in the arms and funnel of both cephalopods investigated so far, which matches expression of these genes in the pedal region of scaphopods, gastropods, and polyplacophorans. Our study suggests that *Hox* genes are expressed in these structures in an evolutionarily conserved fashion and may not have been recruited into the formation of arms and funnel as entirely novel organ systems.

### *ParaHox* genes are expressed in the cephalopod digestive system

Our study also presents for the first-time gene expression patterns of the *ParaHox* genes *Gsx* and *Xlox* in an octopod. While there is a study on *Gsx* in the decapod *Xipholeptos notoides*, no data are known yet for *Xlox* and for *ParaHox* gene expression in octopods overall^38^. In *X. notoides*, *Gsx* is expressed in more brain lobes than in *O. vulgaris*, however, both species express *Gsx* in the lateral lips close to the optic lobes but also in the hindgut (Fig. 13g in Ref.^38^). In contrast to *X. notoides* and numerous other bilaterian species^38^, *O. vulgaris* expresses *Gsx* in the esophagus on a low level, supporting a previous hypothesis that *Gsx* patterned the foregut of the last common bilaterian ancestor^61,62^. *Gsx* expression in the lateral lips of both above-mentioned cephalopod species is reminiscent of neurogenic expression domains such as in the cerebral ganglia of the patellogastropod *Gibbula varia*, the annelid *Platynereis dumerilii* or numerous other bilaterian species^37,38,63^.

## Conclusion

Our study shows that cephalopods exhibit traces of staggered *Hox* expression during early development. This staggered condition can also be observed in homologous body regions of cephalopods and their molluscan relatives. This demonstrates that molecular data still reveal traces of the ancestral molluscan heritage despite all morphological innovations of coleoid cephalopods.

### Supplementary Information

Supplementary Information.

## Data Availability

Raw reads and both resulting transcriptomes are published on Zenodo (https://doi.org/10.5281/zenodo.8136693) and accession numbers on Genbank (Supplementary Table 1).
