# Supplementary material for: Octopod Hox genes and cephalopod plesiomorphies
Source: Sci Rep. 2023 Sep 19;13:15492. doi: 10.1038/s41598-023-42435-0 (PMC10509229; doi:10.1038/s41598-023-42435-0)
Supplement: Supplementary file 1 — Supplementary Information. [file 41598_2023_42435_MOESM1_ESM.pdf]

Octopod *Hox* genes and cephalopod plesiomorphies

Supplementary figures

Cristian Camillo Barrera Grijalba[1], Sonia Victoria Rodríguez Monje[1], Camino Gestal[2], Tim Wollesen[1*]

[1]Department of Evolutionary Biology, Faculty of Life Sciences, University of Vienna, Djerassiplatz 1, 1030 Vienna, Austria.

[2]Institute of Marine Research (IIM-CSIC), Eduardo Cabello 6, 36208 Vigo, Spain.

Email: Tim Wollesen: tim.wollesen@univie.ac.at

Sonia Victoria Rodríguez Monje: sonia.rodriguez@univie.ac.at

*Corresponding author: Tim Wollesen; Email: tim.wollesen@univie.ac.at

Tim Wollesen: Orcid ID: 0000-0003-0464-1254

Sonia Victoria Rodríguez Monje: 0000-0003-2194-4177

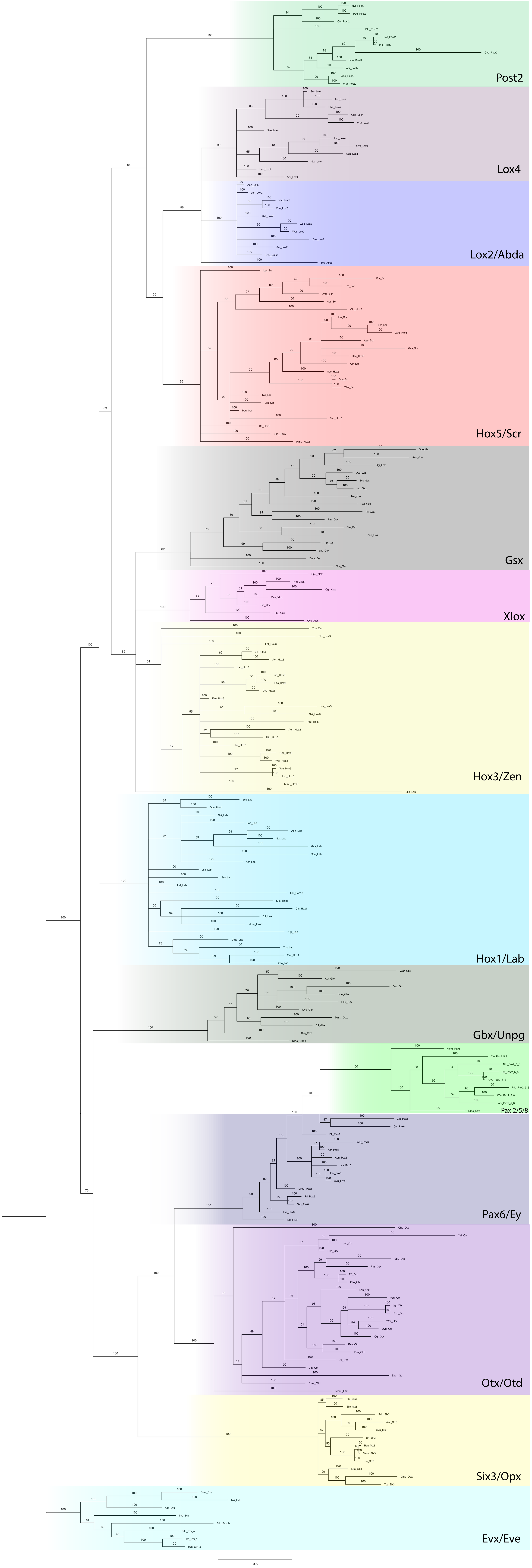
Post2
Lox4
Lox2/Abda
Hox5/Scr
Gsx
Xlox
Hox3/Zen
Hox1/Lab
Gbx/Unpg
Pax 2/5/8
Pax6/Ey
Otx/Otd
Six3/Opx
Evx/Eve
0.8

Figure 1: Orthology analysis of Hox and ParaHox genes from metazoan representatives (Supplementary tables 1, 2) and the putative gene sequences from *Octopus vulgaris* retrieved from the transcriptome. The *even-skipped (Evx/Eve)* subfamily was used as outgroup. Bootstrap values are shown for all the relationships.

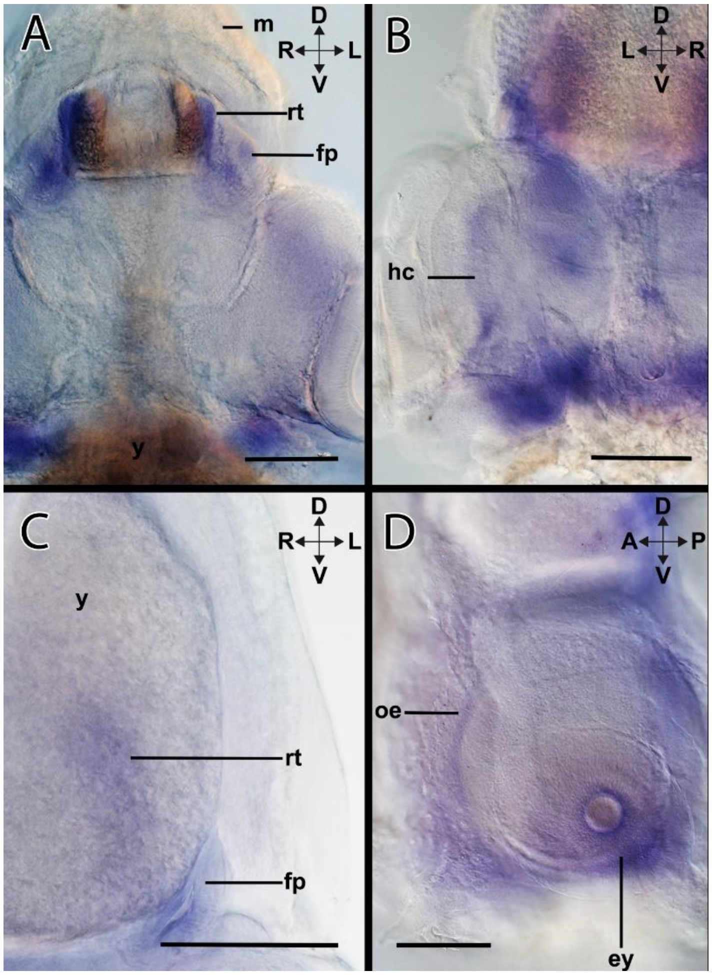

Figure 2: Expression of *Hox1* in developmental stages of *Octopus vulgaris*

Dorsal (D)-ventral (V), anterior (A)-posterior (P), and left (L)-right (R) axes indicate the orientation. Posterior views (A, C), anterior view (B), and lateral view (D). Stage XIV (A-B), Stage XVIII (C-D): **A**: In stage XIV individuals, *Hox1* transcripts can be found in the retractor (rt) in the mantle (m) region, next to the funnel pouches (fp). **B**: From the anterior view, *Hox1* expression domain delimitates the head cover (hc). **C**: In stage XVIII, there is expression of *Hox1* related to the funnel pouches, anterior to the retractor. **D**: In the head region, *Hox1* expression domain surrounds the ocular edges (oe), next to the eye (ey) tissue. Abbreviations: y; yolk. Scale bars: 200µm.

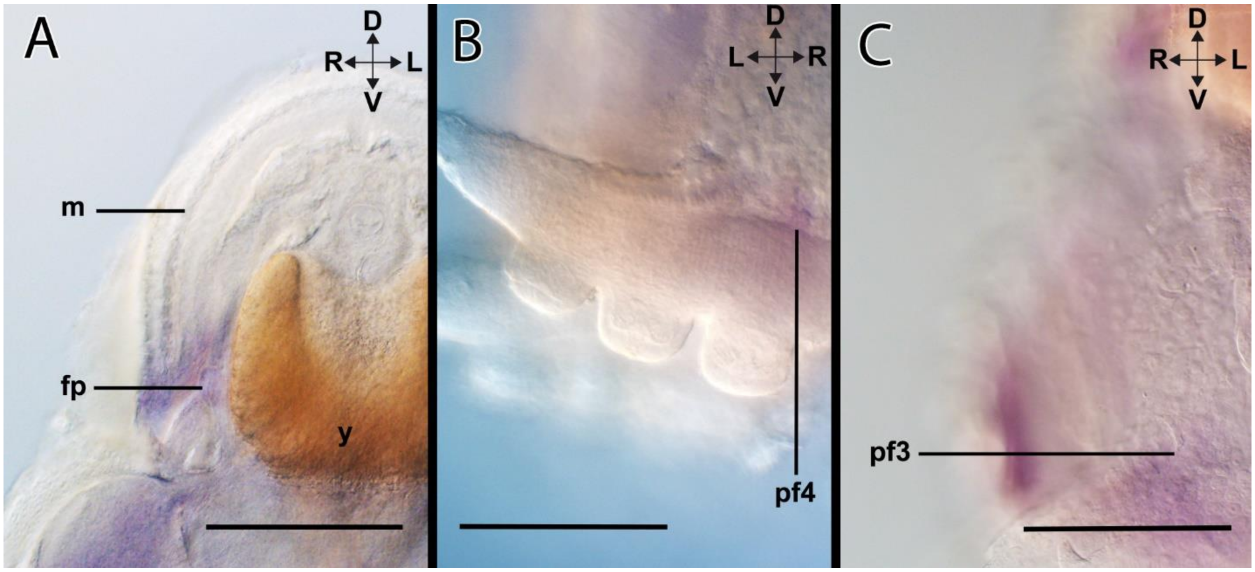

Figure 3: Expression of *Hox3* in developmental stages of *Octopus vulgaris*

Dorsal (D)-ventral (V), anterior (A)-posterior (P), and left (L)-right (R) axes indicate the orientation. Posterior views (A, C). Stage XIV (A-B), Stage XVIII (C): **A**: In stage XIV, *Hox3* transcripts are present in the funnel pouches (fp), in the mantle region (m). **B**: From the anterior view, *Hox3* expression is present in the pillars of the arm pair I (pf1). **C**: In stage XVIII, the expression of *Hox3* involves the pillars of the arm pair III (pf3). Abbreviations: y; yolk. Scale bars: 200µm.

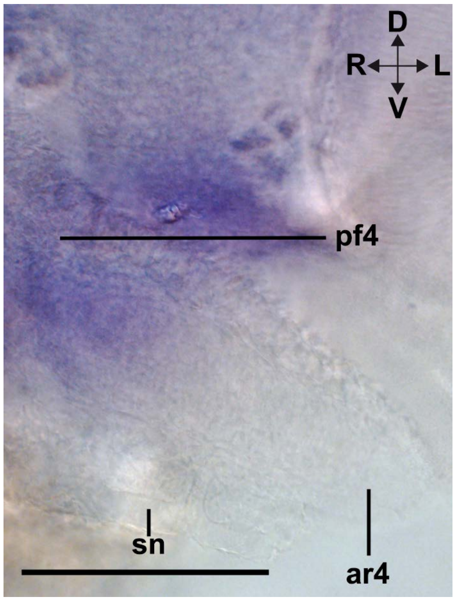

Figure 4: Expression of *Lox4* in developmental stage of *Octopus vulgaris*

Dorsal (D)-ventral (V), anterior (A)-posterior (P), and left (L)-right (R) axes indicate the orientation. Posterior view. Stage XIV: In stage XIV, from the posterior region of the embryo the expression domain of *Lox4* involves the pillar of the arm pair IV (pf4). Abbreviations: sn, sucker; ar, arm. Scale bars: 200μm.

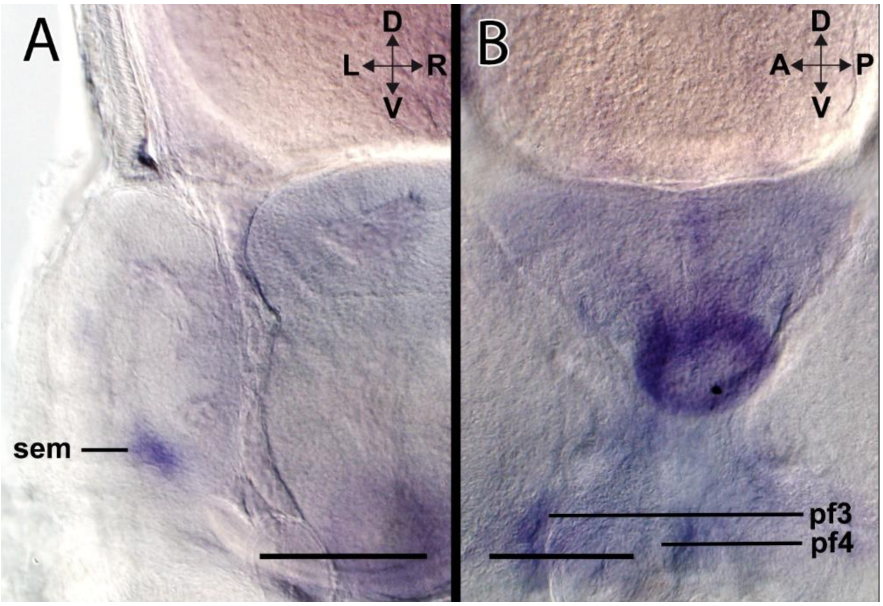

Figure 5: Expression of *Lox2* in developmental stages of *Octopus vulgaris*

Dorsal (D)-ventral (V), anterior (A)-posterior (P), and left (L)-right (R) axes indicate the orientation. Lateral view (A), posterior view (B). Stage XVIII (A-B): A: In stage XVIII individuals, there is expression of *Lox2* in the anterior region of the embryo, close to the supraesophageal mass area (sem). B: From the posterior region, the expression of *Lox2* transcripts is associated with the pillars of the arm pairs III and IV (pf3, pf4). Scale bars: 200µm.

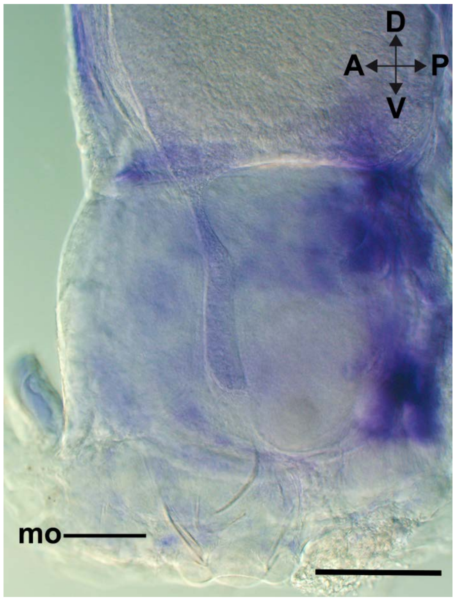

Figure 6: Expression of *Gsx* in developmental stages of *Octopus vulgaris*

Dorsal (D)-ventral (V), anterior (A)-posterior (P), and left (L)-right (R) axes indicate the orientation. Lateral view (A). Stage XVIII (A): In stage XVIII, there is faint *Gsx* transcript expression around the ventral side of the embryo, close to the mouth (mo) area. Scale bars: 200μm.

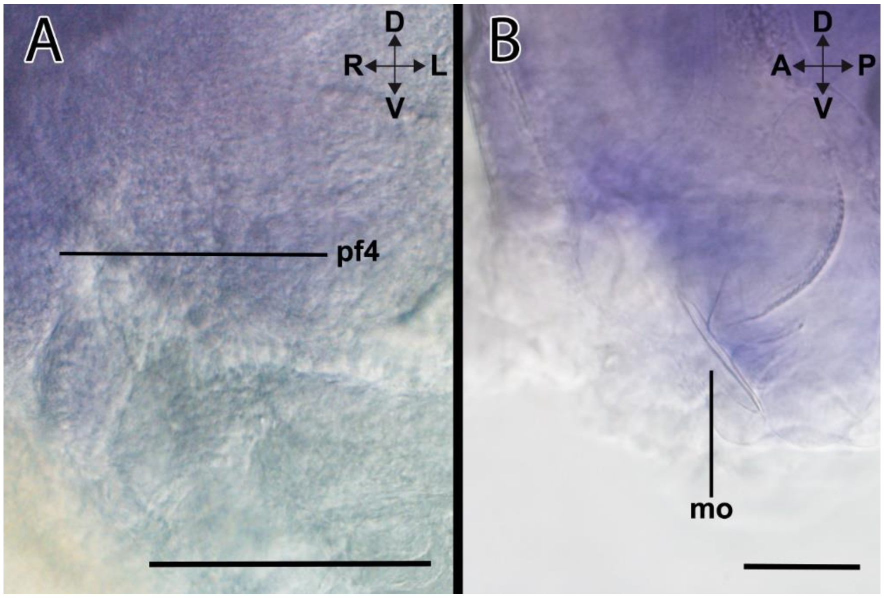

Figure 7: Expression of *Xlox* in developmental stages of *Octopus vulgaris*

Dorsal (D)-ventral (V), anterior (A)-posterior (P), and left (L)-right (R) axes indicate the orientation. Posterior view (A) , lateral view (B). Stage XIV (A), Stage XVIII (B): A: In stage XIV individuals, the expression of *Xlox* is present in the pillars of the arm pair IV (pf4). B: In stage XVIII, *Xlox* can be found in the tissue surrounding the mouth (mo) area. Scale bars: 200µm.

Supplementary tables

Table 1. NCBI accession numbers for the sequences used in the phylogenetic analysis. The currently accepted taxonomic names are in some cases followed by the taxonomically outdated names in parenthesis.

Gene	Organism	Identifiers
Hox1/Lab	*Acanthochitona fascicularis* (*Acanthochitona crinita*)	AKV16302.1
	Antalis entalis	APD15651.1
	Branchiostoma floridae	XP_035657470.1
	Caenorhabditis elegans	NP_498655.1
	Ciona intestinalis	NP_001122333.1
	Drosophila melanogaster	CAB57787.1
	Euprymna scolopes	AAL25804.1
	Flaccisagitta enflata	ABS18809.1
	Steromphala varia (*Gibbula varia*)	ACX84671.1
	Gymnomenia pellucida	APD15663.1
	Lingula anatina	AAD45587.1
	Lithobius atkinsoni	AAL36907.1
	Lineus sanguineus	CAA76295.1

	Lottia cf. kogamogai	APD15692.1
	Mus musculus	NP_034579.3
	Nymphon gracile	ABD46723.1
	Alitta virens (*Nereis virens*)	AAD46166.2
	Nucula tumidula	APD15698.1
	Octopus vulgaris	OR250458
	Sacculina carcini	ABB46347.1
	Saccoglossus kowalevskii	NP_001158384.1
	Symsagittifera roscoffensis	AAN11404.1
	Tribolium castaneum	NP_001107762.1
Hox3/Zen	*Acanthochitona fascicularis* (*Acanthochitona crinita*)	APD15643.1
	Antalis entalis	APD15653.1
	Branchiostoma floridae	XP_035657460.1
	Drosophila melanogaster	NP_476793.1
	Euprymna scolopes	AAR16188.1
	Flaccisagitta enflata	ABS18810.1

	Steromphala varia (*Gibbula varia*)	ADJ18232.1
	Gymnomenia pellucida	APD15665.1
	Haliotis asinina	AAK17185.1
	Xipholeptos notoides (*Idiosepius notoides*)	APD15688.1
	Lingula anatina	AAD45588.1
	Lithobius atkinsoni	AAL36906.1
	Lineus sanguineus	CAA76296.1
	Lottia cf. kogamogai	APD15721.1
	Mus musculus	NP_034582.1
	Alitta virens (*Nereis virens*)	AAD46168.1
	Nucula tumidula	APD15700.1
	Octopus vulgaris	OR250459
	Platynereis dumerilii	ABD04656.1
	Saccoglossus kowalevskii	NP_001158379.1
	Wirenia argentea	APD15711.1
Hox5/Scr	*Acanthochitona fascicularis* (*Acanthochitona crinita*)	AKV16306.1

	Antalis entalis	APD15655.1
	Branchiostoma floridae	ABX39489.1
	Ciona intestinalis	NP_001027665.1
	Drosophila melanogaster	NP_524248.2
	Euprymna scolopes	AAR16189.1
	Flaccisagitta enflata	ABS18812.1
	Steromphala varia (*Gibbula varia*)	ADJ18234.1
	Gymnomenia pellucida	APD15667.1
	Haliotis asinina	ABC00195.1
	Xipholeptos notoides	APD15689.1
	Lingula anatina	AAD45589.1
	Lithobius atkinsoni	AAL36909.1
	Mus musculus	NP_034583.1
	Nymphon gracile	ABD46729.1
	Alitta virens (*Nereis virens*)	AAD46170.1
	Octopus vulgaris	OR250460

	Platynereis dumerilii	ATG29892.1
	Sacculina carcini	AAM50457.1
	Saccoglossus kowalevskii	NP_001158410.1
	Scutopus ventrolineatus	APD15708.1
	Tribolium castaneum	NP_001034523.1
	Wirenia argentea	APD15713.1
Lox4	*Acanthochitona fascicularis* (*Acanthochitona crinita*)	APD15648.1
	Antalis entalis	APD15658.1
	Euprymna scolopes	AAL25810.1
	Steromphala varia	ADJ18237.1
	Gymnomenia pellucida	APD15671.1
	Xipholeptos notoides (*Idiosepius notoides*)	APD15720.1
	Lingula anatina	AAD45593.1
	Lottia cf. kogamogai	KX365132.1
	Nucula tumidula	APD15703.1
	Octopus vulgaris	OR250461

	Scutopus ventrolineatus	APD15710.1
	Wirenia argentea	APD15717.1
Lox2	*Acanthochitona fascicularis* (*Acanthochitona crinita*)	AMT82759.1
	Antalis entalis	APD15657.1
	Steromphala varia (*Gibbula varia*)	ADJ18238.1
	Gymnomenia pellucida	APD15670.1
	Lingula anatina	AAD45592.1
	Alitta virens (*Nereis virens*)	AAD46171.2
	Octopus vulgaris	OR250462
	Platynereis dumerilii	ABD04659.1
	Scutopus ventrolineatus	APD15709.1
	Tribolium castaneum	NP_001034497.1
	Wirenia argentea	APD15716.1
Gsx	*Antalis entalis*	APD15662.1
	Capitella teleta	AAZ23124.1
	Clytia hemisphaerica	ACM62729.1

	Crassostrea gigas	XP_011426138.2
	Euprymna scolopes	AAV85466.1
	Gymnomenia pellucida	APD15675.1
	Homo sapiens	NP_573574.2
	Xipholeptos notoides	APD15691.1
	Lepisosteus oculatus	XP_006627824.1
	Alitta virens (*Nereis virens*)	ABB59695.1
	Octopus vulgaris	OR250463
	Patiria miniata	XP_038057153.1
	Priapulus caudatus	XP_014665932.1
	Ptychodera flava	AAR07642.1
	Zootermopsis nevadensis	XP_021926951.1
Xlox	*Crassostrea gigas*	XP_011426134.2
	Euprymna scolopes	ABD16192.1
	Steromphala varia	ADJ18240.1
	Nucula tumidula	APD15707.1

	Octopus vulgaris	OR250464
	Platynereis dumerilii	ACH87551.1
	Strongylocentrotus purpuratus	NP_999815.2
Otx/Otd	*Branchiostoma floridae*	AAC00193.1
	Caenorhabditis elegans	AAL12002.1
	Ciona intestinalis	NP_001027662.2
	Clytia hemisphaerica	ALJ33545.1
	Crassostrea gigas	XP_011415946.1
	Drosophila melanogaster	NP_001356934.1
	Euperipatoides kanangrensis	CEI71549.1
	Homo sapiens	NP_001186699.1
	Lepisosteus oculatus	XP_006638669.1
	Lingula anatina	XP_013379088.1
	Lottia gigantea	BAQ19208.1
	Mus musculus	NP_001273410.1
	Octopus vulgaris	OR250465

	Patella vulgata	XP_050404761.1
	Patiria miniata	AAP32749.1
	Platynereis dumerilii	CAC19028.1
	Priapulus caudatus	AFY12008.1
	Ptychodera flava	BAA89013.1
	Saccoglossus kowalevskii	NP_001158360.1
	Strongylocentrotus purpuratus	NP_999753.2
	Wirenia argentea	AUG89962.1
	Zootermopsis nevadensis	KDR15576.1
Gbx/Unpg	*Acanthochitona fascicularis* (*Acanthochitona crinita*)	ASM61117.1
	Branchiostoma floridae	XP_035664051.1
	Drosophila melanogaster	NP_477146.1
	Steromphala varia (*Gibbula varia*)	ADJ18239.1
	Mus musculus	NP_034392.1
	Nucula tumidula	ASM61119.1
	Octopus vulgaris	OR250466

	Platynereis dumerilii	CAD43609.1
	Saccoglossus kowalevskii	NP_001158358.1
	Wirenia argentea	ASM61118.1
Evx/Eve	*Homo sapiens*	NP_001291448.1
	Homo sapiens	NP_001073927.1
	Branchiostoma floridae	AAK58953.1
	Branchiostoma floridae	AAK58954.1
	Saccoglossus kowalevskii	NP_001164694.1
	Drosophila melanogaster	NP_523670.2
	Tribolium castaneum	XP_008195064.1
	Capitella teleta	ABG82164.1
Pax6/Ey	*Acanthochitona fascicularis* (*Acanthochitona crinita*)	ARB16026.1
	Antalis entalis	QFU47189.1
	Branchiostoma floridae	CAA11368.1
	Caenorhabditis elegans	NP_001024570.1
	Ciona intestinalis	NP_001027641.1

	Drosophila melanogaster	NP_001014693.1
	Euperipatoides kanangrensis	AGC51117.1
	Euprymna scolopes	AAM74161.1
	Lineus sanguineus	CAA64847.1
	Mus musculus	NP_001231127.1
	Octopus vulgaris	OR250469
	Ptychodera flava	QBZ28543.1
	Saccoglossus kowalevskii	NP_001158383.1
	Wirenia argentea	ARB16028.1
Pax2/5/8	*Acanthochitona fascicularis* (*Acanthochitona crinita*)	ALM30867.1
	Ciona intestinalis	NP_001027652.1
	Drosophila melanogaster	NP_001162834.1
	Xipholeptos notoides (*Idiosepius notoides*)	ALM30869.1
	Mus musculus	NP_035170.1
	Nucula tumidula	ALM30868.1
	Octopus vulgaris	OR250468

	Platynereis dumerilii	AGC12568.1
	Wirenia argentea	ARB16029.1
Post2	*Acanthochitona fascicularis* (*Acanthochitona crinita*)	AKV16308.1
	Crisularia turrita (*Bugula turrita*)	AAS77230.1
	Capitella teleta	ABY67960.1
	Euprymna scolopes	AAL25812.1
	Steromphala varia (*Gibbula varia*)	ACX84674.1
	Gymnomenia pellucida	APD15673.1
	Xipholeptos notoides (*Idiosepius notoides*)	APD15690.1
	Alitta virens (*Nereis virens*)	AAD46176.2
	Nucula tumidula	APD15705.1
	Platynereis dumerilii	ABD04651.1
	Wirenia argentea	APD15719.1
Six3	*Branchiostoma floridae*	XP_035686819.1
	Drosophila melanogaster	AAD39863.1
	Euperipatoides kanangrensis	ABY60729.1

	Homo sapiens	NP_005404.1
	Lepisosteus oculatus	XP_006638703.1
	Mus musculus	NP_035511.2
	Octopus vulgaris	OR250467
	Patiria miniata	XP_038046827.1
	Platynereis dumerilii	CAR66435.1
	Saccoglossus kowalevskii	NP_001158378.1
	Tribolium castaneum	NP_001106938.1
	Wirenia argentea	AUG89963.1

Table 2: Abbreviations of the species names used in the orthology analysis tree.

**Species name**	**Abbreviation**
Acanthochitona fascicularis (formerly erroneously referred to as *Acanthochitona crinita*)	*Acr*
Antalis entalis	*Aen*
Branchiostoma floridae	*Bfl*
Crisularia turrita (*Bugula turrita*)	*Btu*
Caenorhabditis elegans	*Cel*
Capitella teleta	*Cte*
Ciona intestinalis	*Cin*
Clytia hemisphaerica	*Che*
Crassostrea gigas	*Cgi*
Drosophila melanogaster	*Dme*
Euperipatoides kanangrensis	*Eka*
Euprymna scolopes	*Esc*
Flaccisagitta enflata	*Fen*
Steromphala varia (*Gibbula varia*)	*Gva*
Gymnomenia pellucida	*Gpe*
Haliotis asinina	*Has*

Homo sapiens	*Hsa*
Xipholeptos notoides (formerly referred to as *Idiosepius notoides*)	*Ino*
Lepisosteus oculatus	*Loc*
Lineus sanguineus	*Lsa*
Lingula anatina	*Lan*
Lithobius atkinsoni	*Lat*
Lottia cf. kogamogai	*Lko*
Lottia gigantea	*Lgi*
Mus musculus	*Mmu*
Nereis virens (Alitta virens)	*Nvi*
Nucula tumidula	*Ntu*
Nymphon gracile	*Ngr*
Patella vulgata	*Pvu*
Patiria miniata	*Pmi*
Platynereis dumerilii	*Pdu*
Priapulus caudatus	*Pca*
Ptychodera flava	*Pfl*
Saccoglossus kowalevskii	*Sko*

Sacculina carcini	*Sca*
Scutopus ventrolineatus	*Sve*
Steromphala varia	*Sva*
Strongylocentrotus purpuratus	*Spu*
Symsagittifera roscoffensis	*Sro*
Tribolium castaneum	*Tca*
Wirenia argentea	*War*
Zootermopsis nevadensis	*Zne*

Table 3. Sequences of the primers used for the amplification of the cDNA for the synthesis of the RNA-probes. The reverse primers include the sequence of the T7 promoter (in bold). Otherwise, the probe was synthetized using cloning.

Gene	Forward primer sequence	Reverse primer sequence
Hox 1	CGCTATCACTTGTGCGACCC	**TAATACGACTCACTATAGGG**GGCACCTGTTGTTCCTTGAGC
Hox 3	CCGCGGTCGCTCACCA	GATTGAGGACGGTACGGCGA
Hox 5	CCGAACAGTACGACTACTAACG	GAAGGGAGAGGGTCAAGATAGCA
Lox 4	AACGAGAGGGCGGCTTACTG	**TAATACGACTCACTATAGGG**CGGCACAAGGCCAGCAATTC
Lox 2	TGCTACCACCAGCATCGGAG	**TAATACGACTCACTATAGGG**GAAGCTTCCGAGTTTTTATGTGTCT
Gsx	ACGTGTTACCCGCGACATCC	**TAATACGACTCACTATAGGG**TAAGAGTCCCCTTCGTCAGAGG
Xlox	CGCGCTTACATACGCATCAG	**TAATACGACTCACTATAGGG**ATGCCCTTGATTCTGCGAGG
Otx	TATCGGCGGCTACGTCCAAG	**TAATACGACTCACTATAGGG**TTGGTCGTAGTTTTGCGGCG
Gbx	CAAGTCCCCTCGGCCATCTG	**TAATACGACTCACTATAGGG**CCCACCTCACTACAACGGTC
Six3	ACAAACCAAACTACACGTAATCTTC	**TAATACGACTCACTATAGGG**GTCGTTATCGTCGTCGTCGT
Pax6	GCATATGAGCGCCAGTTCCG	**TAATACGACTCACTATAGGG**TCCACCCGGCACCTGTATTG
Pax2/5/8	CGTGCTTAAAAGTGCTGAATGGG	**TAATACGACTCACTATAGGG**TCTGCTCCTGATGGCGTCTG
